# A Dual Perspective of the Action of Lysine on Soybean Oil Oxidation Process Obtained by Combining ^1^H NMR and LC–MS: Antioxidant Effect and Generation of Lysine–Aldehyde Adducts

**DOI:** 10.3390/antiox8090326

**Published:** 2019-08-21

**Authors:** Ana S. Martin-Rubio, Patricia Sopelana, Fumie Nakashima, Takahiro Shibata, Koji Uchida, María D. Guillén

**Affiliations:** 1 Food Technology, Faculty of Pharmacy, Lascaray Research Center, University of the Basque Country (UPV/EHU), Vitoria 01006, Spain; 2 Laboratory of Food and Biodynamic, Graduate School of Bioagricultural Sciences, Nagoya University, Nagoya 464-8601, Japan; 3 Laboratory of Food Chemistry, Graduate School of Agricultural and Life Sciences, The University of Tokyo, Tokyo 113-8657, Japan

**Keywords:** soybean oil, l-lysine, antioxidant, ^1^H NMR, LC–MS, hydroperoxides, aldehydes, epoxides, lysine adducts

## Abstract

Little is still known about both the effect of amino acids on the oxidation course of edible oils and the modifications that the former may undergo during this process. Bearing this in mind, the objective of this work was to study the evolution of a system consisting of soybean oil with 2% of l-lysine under heating at 70 °C and stirring conditions, analyzing how the co-oxidation of the oil and of the amino acid affects their respective evolutions, and trying to obtain information about the action mechanism of lysine on soybean oil oxidation. The study of the oil progress by ^1^H Nuclear Magnetic Resonance (^1^H NMR) showed that the presence of lysine noticeably delays oil degradation and oxidation products generation in comparison with a reference oil without lysine. Regarding lysine evolution, the analysis by ^1^H NMR and Liquid Chromatography–Mass Spectrometry of a series of aqueous extracts obtained from the oil containing lysine over time revealed the formation of lysine adducts, most of them at the ε position, with n-alkanals, malondialdehyde, (*E*)-2-alkenals, and toxic oxygenated α β-unsaturated aldehydes. However, this latter finding does not seem enough to explain the antioxidant action of lysine.

## 1. Introduction

The antioxidant ability of proteins and peptides [1] has long generated great interest among food scientists, especially in the last two decades, when numerous studies have been conducted to investigate the potential of protein hydrolysates of diverse origin as antioxidants in food systems [1,2,3]. In this context, the monitoring of lipid oxidation is usually carried out by means of classical methodologies such as peroxide value, conjugated dienes, and/or TBARS (2-Thiobarbituric Acid Reactive Substances) assay, in some cases combined with the determination of volatile aldehydes like propanal or hexanal. However, in most of the studies a global view of the oxidation process is not achieved, and this hinders the intricate task of delving into antioxidant mechanisms.

It is also noteworthy that most of these studies mainly focus on the evolution of the lipid matrix, and little attention is given to the changes provoked by oxidation in amino acids. In fact, only a reduced number of the multiple works dealing with the antioxidant effect of proteins, peptides, or amino acids on food systems combine the simultaneous monitoring of both the lipid oxidation process and the modifications suffered by amino acids [2,3]. Among these latter, the formation of carbonyl groups from basic amino acids, the oxidation of free thiols to give disulfide bridges, or the hydroxylation of aromatic amino acids should be mentioned. Protein oxidation can also lead to cleavage of the polypeptide chain and to formation of cross-linked protein aggregates [4]. Furthermore, functional groups of proteins can react with lipid oxidation products like hydroperoxides or aldehydes [5,6].

Various mechanisms have been proposed to explain the antioxidant action of amino acids and proteins, such as binding of metal ions or free radical scavenging ability [1,7,8], which could reduce the concentration in the medium of ions like Fe^2+^ and Cu^2+^, and/or of free radicals, both of which can accelerate lipid oxidation. In addition, the ability of some amino acids like methionine to reduce lipid hydroperoxides to hydroxides has also been considered as an antioxidant mechanism by interfering with the propagation of lipid oxidation [1]. However, the specific pathways through which amino acid residues or free amino acids are able to affect the course of lipid oxidation require further investigation, for a better understanding of their antioxidant effect.

Another issue worth mentioning is related to the potential of free amino acids as antioxidants in food systems. Unlike what can happen in proteins, where certain antioxidant amino acids can be buried into the protein structure, free amino acids are directly available to take part in different types of reactions, and this might enhance their antioxidant action. Despite this, studies aimed at investigating the effect of free amino acids on the oxidative stability of food lipids [7,9,10,11] are comparatively less abundant than those concerning proteins and their hydrolysates [1].

Taking into account all the above, this work addresses the effect of L-lysine on the oxidation process of soybean oil, analyzing how the co-oxidation of the oil and of the amino acid affects their respective evolutions. Given that the antioxidant effect of lysine on soybean oil under very similar conditions to those used in this study was already proved in a previous work [11], interest has been focused on the evolution of the amino acid; notwithstanding, the progress of the oil during oxidation has also been monitored in order to establish relationships between both processes. It must be noticed that the co-oxidation of amino acids and proteins is awakening increasing awareness due to its biological and health implications [12].

To achieve the aims of this study, refined soybean oil and the same oil containing 2% in weight of lysine were heated under continuous stirring in a magnetic stirrer at 70 °C. The oil progress was monitored by ^1^H Nuclear Magnetic Resonance (^1^H NMR), while the changes in lysine were studied by Liquid Chromatography followed by Mass Spectrometry (LC–MS) and also by ^1^H NMR, in order to explore the potential of this latter technique to follow the evolution of lysine under oxidative conditions. Thus, the outcomes of this work might contribute to gaining further insight into the co-oxidation of lipids and amino acids, and to some extent of lipids and proteins; for this purpose a food system which would be feasible in the context of human nutrition has been studied from a dual perspective: oil and amino acid, since it is expected that there is a relationship between both evolutions, which might provide information about the action mechanism of lysine on soybean oil.

## 2. Materials and Methods

### 2.1. Samples

The original samples were a commercial refined soybean oil (RSO), containing all its original components, purchased from a multinational company, and the same oil enriched with 2% by weight of l-lysine (RSO + 2LYS). The molar percentages of the different types of oil acyl groups were determined by ^1^H NMR, as in previous works [13], employing equations [S1–S4] (see supplementary material). These were 6.3 ± 0.1 for linolenic, 49.5 ± 0.1 for linoleic, 25.5 ± 0.3 for oleic, and 18.7 ± 0.3 for saturated groups. The l-lysine used had a purity ≥98% and was purchased from Sigma-Aldrich (St. Louis, MO, USA). Due to the difficulty of uniformly distributing lysine into the oil, caused by the insolubility of this amino acid in this nonpolar medium [14], RSO + 2LYS samples were prepared by directly weighing 8 g of RSO in a beaker and then adding 2% by weight of lysine.

### 2.2. Oxidation Process 

Samples of RSO and of RSO + 2LYS (8 g of oil in all cases), prepared in beakers of 5 cm diameter, one per day of sampling, were placed on a multiple magnetic stirrer heated at 70 °C and stirred at 180 rpm with magnets of 4.5 cm long. The magnetic stirrer was devoid of any coverage, so the oil samples were exposed to light during the day. Samples submitted to oxidative conditions for different periods were taken throughout the oxidation process for their study, until the stirring magnet stopped rotating due to the great viscosity increase provoked by the total polymerization of the oil [15]. The oxidation process was carried out in duplicate, in order to obtain average values for all the compounds studied.

#### 2.2.1. Monitoring by ^1^H NMR of the Evolution of RSO and of RSO + 2LYS Samples throughout the Oxidation Process

The ^1^H NMR spectra of both the original RSO and RSO + 2LYS samples, and of these samples after their being submitted to oxidative conditions over different periods of time were acquired using a Bruker Avance 400 spectrometer operating at 400 MHz, following the same procedure as in previous works [13]. Experimental details and information about acquisition parameters are given in the supplementary material.

The molar percentages of the two kinds of polyunsaturated acyl groups present in soybean oil and the concentrations of the oxidation products generated were estimated throughout the oxidation process from the ^1^H NMR spectra, on the basis of the ^1^H NMR spectral signal assignments shown in Table S1 (see supplementary material). This contains bibliographic data and data coming from several standard compounds, also indicated in the supplementary material. The determination of the molar percentages of linolenic and linoleic acyl groups was done using Equations [S1] and [S2] (see supplementary material), respectively. Regarding oxidation compounds, their concentrations were estimated by means of Equation [S5] (see supplementary material). 

#### 2.2.2. Extraction of Lysine and Some of its Derivatives from the RSO + 2LYS Sample after Being Submitted for Different Periods of Time to Oxidative Conditions

Lysine and some of its derivatives formed during the oxidation process were extracted from the corresponding RSO + 2LYS sample as follows; 0.16 g of each sample was poured into a 1.5 mL Eppendorf microtube and mixed with 800 μL of the extraction solvent. Several deuterated solvents were tested to extract the amino acid and its derivatives from the system and to study their recovery by means of ^1^H NMR: water, water with different methanol percentages, methanol, and acid water (0.5 M of HCl, pH close to 1.5). The best extraction efficiency was achieved using acid water, so this was the solvent chosen. After adding the solvent, each Eppendorf microtube was shaken for 10 min with an automatic tube stirrer and then centrifuged at 10,000 rpm for another 10 min. The aqueous phase was taken out with a pipette, filtered through a 0.45 µm filter (GL Science Inc., Tokyo, Japan) using a 1 mL syringe (Terumo corporation, Tokyo, Japan), and poured into another Eppendorf microtube. The extracts were analyzed both by LC-MS and by ^1^H NMR. It must be pointed out that milliQ water was used to obtain the extracts intended for the LC–MS analysis, while deuterated water was used for ^1^H NMR.

##### Study of the Extracts by LC-MS

The aqueous extracts obtained from the RSO + 2LYS sample throughout the oxidation process were studied by LC–MS. However, due to analytical requirements, in this case it was necessary to add milliQ water to these extracts before their analysis, in order to make the pH less acid (near 2.0). The LC–MS analysis conditions, together with the procedures followed for the identification and quantification of lysine and its derivatives are given in the supplementary material.

The evolutions of lysine and its derivatives were monitored only up to the 22nd day of the oxidation process; afterwards, the oil polymerization degree hindered the extraction of lysine and lysine derivatives in the aqueous phase, so it was not possible to obtain extracts directly comparable to the previous ones. 

##### Study of the Extracts by ^1^H NMR

The aqueous extracts obtained from the RSO + 2LYS sample throughout the oxidation process were also studied by ^1^H NMR. The procedure followed was the same as for the lipid samples (see supplementary material) but, in this case, 600 μL of the aqueous extract (see Section 2.2.2) was taken directly from the Eppendorf microtube and placed in an NMR tube for analysis.

Identification of the compounds in these extracts was based on the assignment of the ^1^H NMR spectral signals made using standard compounds. To this aim, L-lysine, Nε-formyl-lysine, Nα-acetyl-lysine, and Nε-acetyl-lysine were purchased from Cymit Quimica (Barcelona, Spain). The chemical shifts, multiplicities, and assignments of their signals are given in Table S2 (supplementary material). 

The quantification of lysine and its derivatives by ^1^H NMR constitutes one of the goals of this work, so the procedure developed for this purpose will be described below, in the Results and discussion section.

## 3. Results and Discussion

### 3.1. Effect of the Presence of Lysine on Soybean Oil Evolution. Monitoring by ^1^H NMR

Given that an exhaustive analysis of the effect of different lysine proportions on the evolution of refined soybean oil under very similar conditions to those in this study was performed before [11], in the present work a less detailed discussion will be carried out; however, it is considered necessary to include these data in order to relate oil evolution to that of lysine.

#### 3.1.1. Effect on the Degradation Rate of Polyunsaturated Acyl Groups

**Figure 1:**
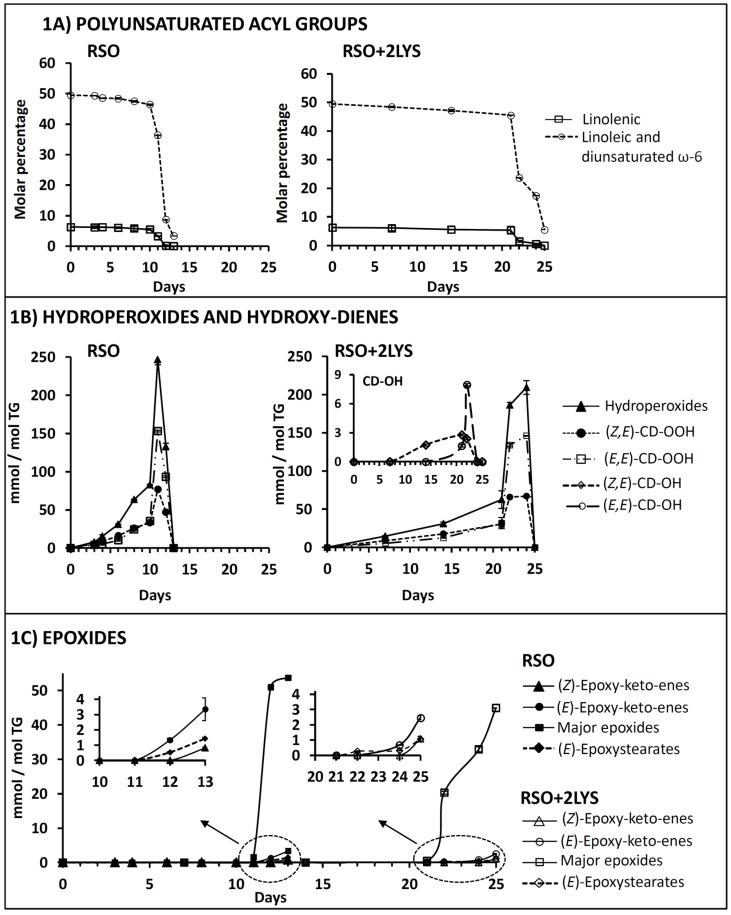
Evolution throughout the oxidation process, in the reference oil (RSO) and in the sample with lysine added (RSO + 2LYS), of: (**A**) the molar percentages of linolenic and linoleic acyl groups; (**B**) the concentrations, in mmol/mol TG, of hydroperoxides and their associated conjugated (*Z,E*)- and (*E,E*)-dienes ((*Z,E*)- and (*E,E*)-CD-OOH), and of conjugated (*Z,E*)- and (*E,E*)-hydroxy-dienes ((*Z,E*)- and (*E,E*)-CD-OH); and (**C**) the concentrations, in mmol/mol TG, of different kinds of epoxides. All the figures reported are mean values. RSO: refined soybean oil. 2LYS: 2% by weight of l-lysine. TG: triglyceride.

The degradation of soybean oil can be estimated from the evolution of the molar percentages of the two types of polyunsaturated acyl groups present in this oil (linolenic and linoleic), which is represented versus time in days in Figure 1A. As this graph shows, the evolution of polyunsaturated groups splits into two very distinct stages, characterized by markedly different degradation rates. The addition of lysine greatly lengthened the first stage, during which acyl groups degraded much more slowly, provoking an important slowdown in oil degradation; consequently, it was not until day 22 that a sudden decrease of the molar percentages of polyunsaturated groups occurred, whereas in RSO this took place for day 11. It was also noticeable that the degradation extent of linoleic groups at the end of the oxidation process in the lysine-enriched sample was somewhat lower than in the original oil. These results indicated that lysine exerts a clear antioxidant effect and it is to be expected that this will also affect the formation of oxidation compounds such as hydroperoxides, epoxides, and aldehydes.

#### 3.1.2. Effect on Hydroperoxide Formation 

As a consequence of acyl group degradation, hydroperoxides giving ^1^H NMR signals between 8.3 and 9.0 ppm (see Table S1) were generated. The evolution of the concentration of these hydroperoxides and of their associated (*Z,E*)- and (*E,E*)-conjugated dienes, expressed in millimoles per mole of triglyceride (mmol/mol TG), is shown in Figure 1B. This shows that, in agreement with observations made in the degradation of linolenic and linoleic acyl groups (see Figure 1A), hydroperoxide concentration rose at a much higher rate in RSO than in RSO + 2LYS, reaching in the first case its maximum concentration on day 11 (247 mmol/mol TG), and on day 24 in the second (210 mmol/mol TG).

This suggested that lysine could be acting as a free radical scavenger, in line with the findings of Xu, Zheng, Zhu, Li, and Zhou [8]. According to this hypothesis, it might interact with radicals coming from polyunsaturated acyl groups and/or from the earliest formed hydroperoxides, thus limiting the propagation stage of the radicalary reaction, to the point when it no longer seems possible to slow down the oxidation course, and hydroperoxide accumulation occurs. Indeed, in the extensive review of the reactions between proteins and oxidized lipids carried out by Schaich in 2008 [16], it is stated that, in systems containing lipids and proteins, radical transfer from lipids to proteins occurs early in lipid oxidation, and this provokes an antioxidant effect on lipids.

If hydroperoxides with (*Z,E*)- and (*E,E*)-conjugated-dienes ((*Z,E*)- and (*E,E*)-CD-OOH) are monitored separately from their respective signals “d” and “c”(see Table S1), it can be observed in Figure 1B that, in agreement with findings for total hydroperoxides, the addition of lysine delayed the concentration increase of both types of CD-OOH. However, it did not affect either their maximum concentrations or their relative proportions throughout time when compared with the reference oil.

#### 3.1.3. Effect on Conjugated (*Z,E*)- and (*E,E*)-Hydroxy-Diene Formation 

In accordance with previous findings [11], in RSO + 2LYS submitted to oxidative conditions, very small signals that could be tentatively assigned to (*Z,E*)- and (*E,E*)-hydroxy-dienes ((*Z,E*)- and (*E,E*)-CD-OH, signals “b” and “e” in Table S1) appeared between days 14 and 22. However, these signals were not perceived in any of the spectra of RSO. As can be observed in the enlargement of Figure 1B, the maximum concentrations of (*Z,E*)- and (*E,E*)-CD-OH were reached after 21 and 22 days, respectively. Therefore, the formation of hydroxy-dienes could be due to the ability of lysine to reduce hydroperoxides to more stable hydroxides, and this would contribute to its global antioxidant effect, since this reaction avoids to a certain extent the decomposition of hydroperoxides to give other types of more reactive oxidation products.

#### 3.1.4. Effect on Epoxide Formation

The addition of 2% of lysine to RSO delayed the appearance of the different types of epoxides monitored to a remarkable extent. This can be observed in Figure 1C, which displays the respective evolutions of the concentrations of: (i) the so-called major epoxides, which include various classes of epoxides giving signals at 2.9 and 3.1 ppm approximately (see Table S1); (ii) (*Z*)- and (*E*)-epoxy-keto-enes, presumably derived from polyunsaturated groups (see signals “i”, “j1”, and “j2” in Table S1), and; (iii) (*E*)-epoxystearates, generated from oleic groups (see signal “f” in Table S1). Moreover, the concentration increase of major epoxides over time was also somewhat slower in the RSO + 2LYS sample, in such a way that the maximum reached in this system (44.92 mmol/mol TG) was slightly lower than in the original oil (53.66 mmol/mol TG).

#### 3.1.5. Effect on Aldehyde Formation and on Their Concentration Evolution

**Figure 2:**
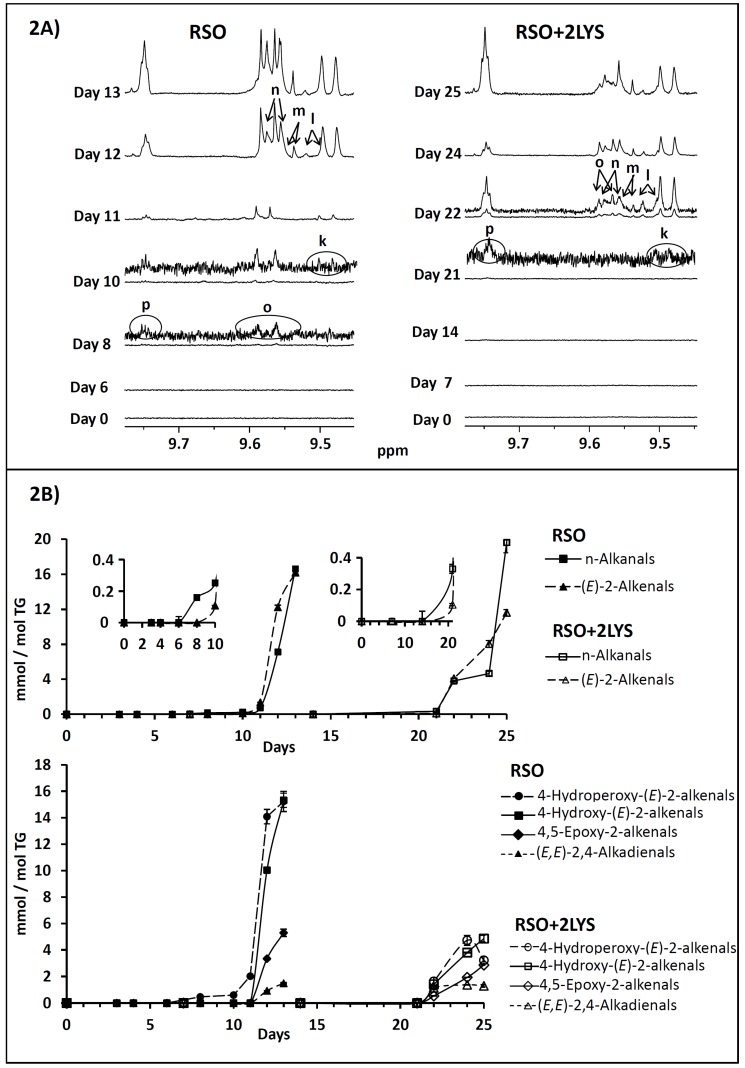
Evolution throughout the oxidation process, in the reference oil (RSO) and in the sample with lysine added (RSO + 2LYS), of: (**A**) the ^1^H NMR spectral signals of aldehydes: *n*-alkanals (signal “p”), 4-hydroperoxy-(*E*)-2-alkenals (signal “o”), (*E*)-2-alkenals (signal “k”), 4-hydroxy-(*E*)-2-alkenals (signal “n”), 4,5-epoxy-2-alkenals (signal “m”), and (*E,E*)-2,4-alkadienals (signal “l”); and (**B**) their respective concentrations, in mmol/mol TG, given as mean values. Letters in Figure (**A**) agree with those in Table S1, and all the plots have been drawn at a fixed value of absolute intensity to be valid for comparative purposes. RSO: refined soybean oil. 2LYS: 2% by weight of l-lysine. TG: triglyceride.

The appearance and evolution of aldehydic signals in the ^1^H NMR spectra of the oil samples throughout the oxidation process is shown in Figure 2A, and the evolution of their concentrations, expressed in mmol/mol TG, in the graphs displayed in Figure 2B.

The addition of lysine to soybean oil affects aldehyde evolution somewhat differently than the rest of oxidation products above-mentioned since, in addition to a notable delay in aldehyde generation (from day 8 in RSO to day 21 in RSO + 2LYS, see Figure 2A), it also caused a pronounced reduction in the concentration of the different types of oxygenated α,β-unsaturated aldehydes (letters “m”, “n”, and “o” in Figure 2A). A diminution in the level of (*E*)-2-alkenals (see Figure 2B) was also observed in the oil containing lysine, although not so striking as in the case of the aldehydes previously mentioned. Therefore, given that the ability of lysine to react with aldehydes is well-known [6], the marked decrease observed in the concentration of aldehydes, especially in the case of the most reactive ones like 4-hydroperoxy- and 4-hydroxy-(*E*)-2-alkenals [17], could be due to their reaction with lysine.

It is worth noticing that, unlike unsaturated aldehydes, the concentration of n-alkanals did not decline but rather slightly increased in the sample containing lysine, in comparison with the reference oil (see Figure 2B). In this sense, saturated aldehydes could proceed from pyrrolization reactions of lysine with epoxy-alkenals like 4,5-epoxy-decenal [18]. Notwithstanding, the occurrence of other types of reactions, leading to the generation of saturated aldehydes should not be discarded either.

Finally, it only remains to add that during the oxidation process of RSO, a singlet at near 8 ppm appeared in the ^1^H NMR spectrum, simultaneous to the appearance of aldehyde proton signals, whose intensity increased parallel to that of aldehydes. This singlet has been tentatively attributed to the proton of the hemiacetal group, formed from aldehydes [19]. It is noteworthy that this signal did not appear in the RSO + 2LYS spectra, maybe because the presence of lysine could provoke competitive reactions with aldehydes that hinder the formation of hemiacetals.

### 3.2. Analysis of the Changes that Lysine Might Undergo or the Reactions in Which it Could be Involved during the Oxidation Process of the RSO + 2LYS Sample

#### 3.2.1. Decrease in Lysine Concentration 

**Figure 3:**
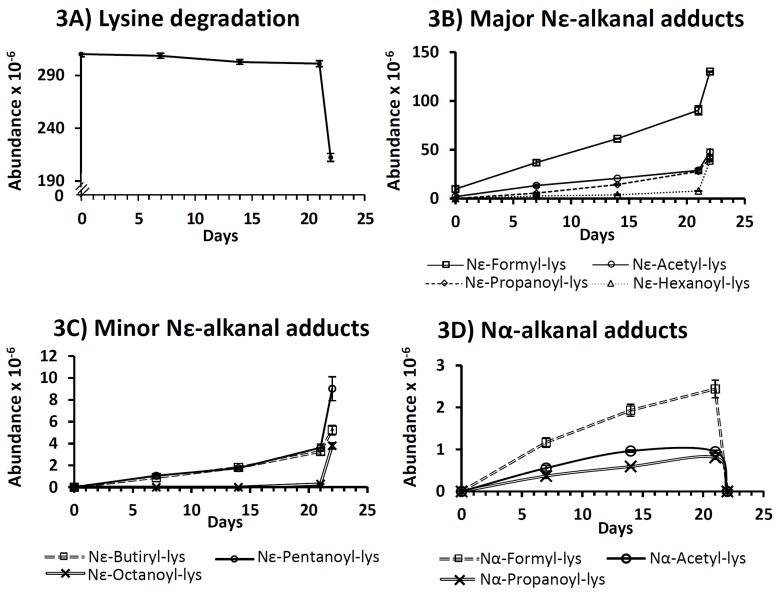
Evolution of the abundances in the aqueous extracts of the RSO + 2LYS oil sample obtained throughout the oxidation process, determined by LC–MS, of the base peaks of: (**A**) lysine; (**B**) major Nε-lysine-alkanal adducts; (**C**) minor Nε-lysine-alkanal adducts; and (**D**) Nα-lysine-alkanal adducts. All the figures reported are mean values. Lys: lysine.

This was monitored by LC–MS through the measurement of the abundance of its mass spectrum base peak in the several aqueous extracts obtained from the RSO + 2LYS sample until day 22, since beyond this sampling time the state of the sample did not allow one to obtain extracts under comparable conditions (see Section 2.2.2). As Figure 3A shows, the abundance of lysine decreased only slightly during most of the oil oxidation process until day 22, when it exhibited a sharp decrease. This coincides with the moment when oil degradation became very fast, in such a way that hydroperoxides were close to their maximum concentration and the levels of all the secondary oxidation products monitored began to increase (see Figure 1 and Figure 2).

#### 3.2.2. Metal Chelation Reactions

**Figure 4:**
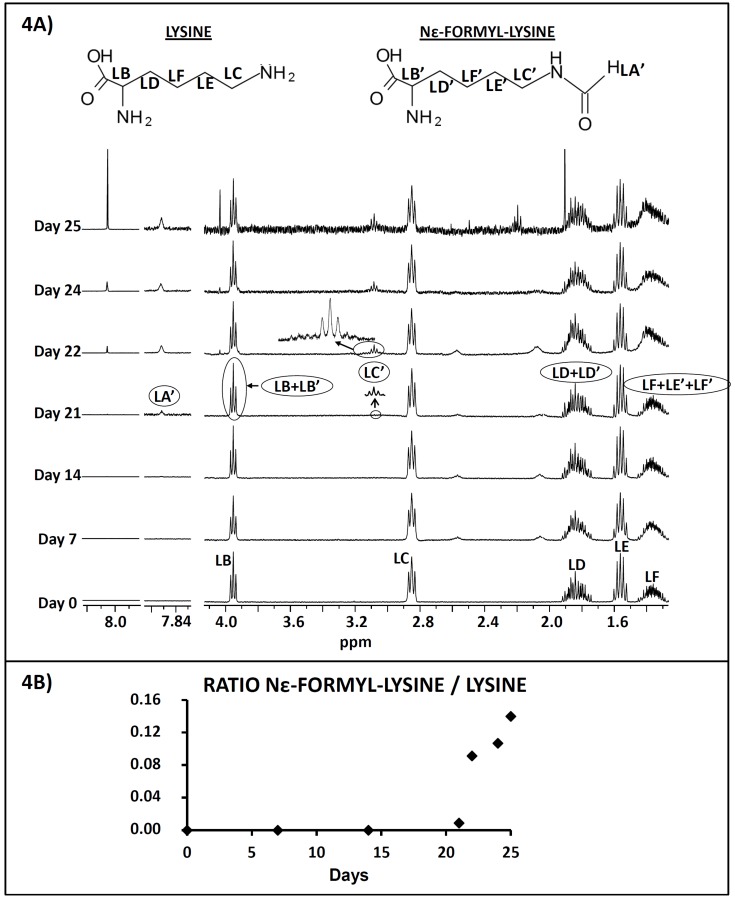
(**A**) ^1^H NMR spectrum of the aqueous extract of the RSO + 2LYS sample before being heated (day 0), and its evolution throughout the oxidation process. Letters agree with those in Table S2. All the plots have been drawn at a fixed value of absolute intensity to be valid for comparative purposes. (**B**) Evolution of the ratio between the molar concentrations of formyl-lysine and lysine, determined by ^1^H NMR, in the aqueous extracts of the RSO + 2LYS sample obtained throughout the oxidation process. All the figures reported are mean values.

Lysine has been shown to exhibit the ability to bind metals [8,20]. Regarding this issue, changes in the ^1^H NMR spectrum of lysine might occur due to its hypothetical interaction with metals present in the system, since according to some authors [20,21] this can provoke the broadening of some ^1^H NMR lysine signals. However, as can be observed from Figure 4A, which shows the ^1^H NMR spectrum of the aqueous extract of the RSO + 2LYS sample before heating (day 0) and its evolution throughout the oxidation process, this did not occur in our work. Hence, a metal-binding effect of lysine cannot be inferred from this study.

#### 3.2.3. Oxidation Reactions Yielding α-Aminoadipic Semialdehyde

In the presence of reactive oxygen species and transition metals such as iron and copper, an oxidative deamination of lysine can occur, leading to the formation of α-aminoadipic semialdehyde (AAS) [22]. Although this compound has been regarded as one of the most abundant carbonyl products of metal-catalyzed oxidation of proteins in food and biological systems [23], it has not been detected either by LC–MS or by ^1^H NMR in this study. This could be due, among other factors, to the oxidative conditions used in our investigation and/or to the composition of the system.

#### 3.2.4. Reactions with Lipid Oxidation Products

In addition to other oxidative changes, the simultaneous oxidation of lipids and amino acids can induce the reaction of the latter with lipid oxidation products. Among these, the available scientific literature mainly focuses on reactions with aldehydes of varying natures and, to a much lesser extent, with hydroperoxides; however, only very few studies can be found concerning other types of oxidation products, as will be commented below.

##### Reaction with Hydroperoxides

Although there are some studies dealing with the reaction of hydroperoxides with proteins [24,25], to the best of our knowledge, little is known about the structure of the products that can be generated as a consequence of such reactions. In this regard, the ability of lysine to react with linoleic group hydroperoxides, giving rise to an amide-type adduct designated as Nε-hexanoyl-lysine, has been suggested by some researchers [26]. This compound was detected by LC–MS in the aqueous extract of the RSO + 2LYS sample after 7 days under oxidative conditions (see Figure 3B), and its abundance exhibited an increase with time, especially at the end of the monitoring period. However, as far as we know, the exact mechanism leading to the generation of this compound from hydroperoxides has not been described.

Despite the lack of information related to compounds resulting from the reaction of lysine and hydroperoxides, it has been reported that this class of oxidation products can mediate covalent modifications of lysine by saturated aldehydes, giving rise to N-alkanoyl (amide type) lysine adducts, while hydroperoxides are reduced to alkyl-hydroxides [27]. Therefore, considering that, as mentioned above, hydroxy-dienes have been detected only throughout the oxidation process of the sample RSO + 2LYS (see Section 3.1.3), this might be evidencing the occurrence of reactions between hydroperoxides and lysine.

##### Reaction with Epoxides

Even when epoxides are considered very reactive and toxic compounds [16], little is known about their reactions with proteins or amino acids. In this regard, Pokorny, Klein, and Koren [28] observed the binding of 9,10-epoxystearic acid to albumin, but the structure of the compound generated was not identified. However, in later studies the formation of aminols derived from this type of reaction was reported by Lederer [29]. With respect to lysine, its reaction with long-chain epoxy-keto-ene fatty acids exhibiting a 4,5-epoxy-1-keto-2-pentene system to give long-chain pyrrole fatty esters has been observed [30].

Although neither the just-mentioned types of pyrrole derivatives nor aminols have been identified by any of the analytical techniques used in this work, as Figure 1C shows, a slightly lower level of epoxides was noticed at the end of the oxidation process in the RSO + 2LYS sample than in RSO. Therefore, although this could be in part attributable to the later detection of epoxides in the oil enriched with lysine and to their slower increase with time before total oil polymerization was reached, the possible reaction of epoxides with lysine should not be discarded. 

##### Reaction with Saturated Aldehydes

Aldehydes are by far the most extensively studied of all the different types of lipid oxidation compounds in terms of their reactivity towards proteins, peptides, and amino acids, and indeed the ability of lysine to take part in such reactions has already been described [6,16,27].

Some lysine adducts with saturated aldehydes, all of them of the amide type, were identified by means of LC–MS in the aqueous extracts of the RSO + 2LYS sample from day 7 onwards. This finding would support the involvement of hydroperoxides in the generation of this type of adducts, mentioned in Section 3.2.4, and their role in the generation of hydroxy-dienes (see Section 3.1.3). The identification of these adducts was carried out, as described in the supplementary material, by comparison of their mass spectra with those of the n-alkanal adducts synthesized in the laboratory. Their respective retention times (RT), base peaks (BP), and other mass spectra fragments obtained with both cone potentials 20 and 35 V, are shown in Table 1. It can be observed that, although adducts at both the Nε and Nα positions were detected, the former were more numerous than the latter, which evidences the higher reactivity of the Nε position of lysine.

Regarding the Nε-adducts, the mass spectra obtained with cone potential 35 V (see Table 1) exhibited three rupture fragments characteristic of lysine: 84 (loss of the –NH_2_ group and of the –COOH group at the α-position), 130 (loss of –NH_2_ at ε-position) and 147 (molecular weight of lysine+1) [31]. In addition, another distinctive fragment was also observed (underlined in Table 1), which coincides with the molecular weight of the corresponding adduct subtracting the amino and the carboxylic groups supported on the α-carbon; this was not observed in the Nα-adducts, where the only fragment found in the spectra obtained with cone potential 35 V, apart from that of the base peak, was that with *m*/*z* 84.

Evolutions of the abundances of the mass spectra base peaks of each of the above-mentioned derivatives during the RSO + 2LYS sample oxidation are shown in Figure 3 (Figure 3B–D). As can be observed from these graphs, the abundances of the several lysine adducts increased with time; this growth was particularly noticeable at the end of the oxidation process (between days 21 and 22), when lysine concentration showed a steep decline (see Figure 3A), and it took place alongside the generation of most aldehydes in the RSO + 2LYS sample (see Figure 2A). Among the various adducts detected, the one due to the reaction of lysine with formaldehyde (Nε-formyl-lysine) exhibited the highest abundance (see Figure 3B), followed by lysine adducts with acetaldehyde (Nε-acetyl-lysine), propanal (Nε-propanoyl-lysine), and hexanal (Nε-hexanoyl-lysine). These findings revealed either that the adducts with the lowest molecular weight aldehydes are the most profusely generated, in agreement with Benedetti and Comporti [32], or that these are more water-soluble, and so better extracted, than those having a higher number of carbon atoms; notwithstanding, both factors might even simultaneously influence the results obtained. 

Regarding Nε-hexanoyl-lysine, it is worth noticing that, as commented in Section 3.2.4, this compound might also derive from the direct reaction of lysine with hydroperoxides [26]. However, considering that the abundance of this adduct only increased considerably at the end of the monitoring period (see Figure 3B), it seems more plausible to think of hexanal as the compound mainly involved in the generation of this adduct [27].

Furthermore, Nε-lysine adducts with butanal, pentanal, and octanal were also detected, though in lower abundances than those of the previously mentioned compounds (see Figure 3C). It is worth noticing that despite nonanal being generated in higher abundance than octanal throughout the oxidation process of soybean oil (data not shown), adducts with the former have not been detected; this would reinforce all the aforementioned regarding the influence of both aldehyde reactivity and water solubility of the adducts on the results obtained.

Finally, as mentioned above, Nα-adducts were also found from day 7 to day 21, but in very small abundances (see Figure 3D).

All the previously mentioned adducts, except that with octanal were detected from day 7 onwards (see Figure 3B–D), although saturated aldehydes were not noticed by ^1^H NMR in the RSO + 2LYS sample until day 21 (see Figure 2A). In this sense, it must be taken into account that, despite not being detected by ^1^H NMR, aldehydes in small concentrations could have been present in the RSO + 2LYS sample, either due to their generation in the earliest stage of hydroperoxide decomposition or as components of the starting soybean oil.

If the outcomes of the analysis of the aqueous extracts of the RSO + 2LYS sample are compared with those coming from the oil phase, it appears striking that, despite the generation of several adducts between lysine and *n*-alkanals, the final concentration of this type of aldehydes in the RSO + 2LYS sample was not lower than in the nonenriched oil (see Figure 2B). In this sense, considering that the adducts generated between lysine and n-alkanals of low molecular weight, like formaldehyde, acetaldehyde, or propanal were the most abundant, it could be thought that lysine mainly forms adducts with very volatile aldehydes, which may tend to escape from the liquid oil matrix in the sample RSO. Therefore, it might be thought that this type of aldehyde would make a minor contribution to the total of n-alkanals detected in RSO. However, as mentioned in Section 3.1.5, other factors might also be involved in the results observed.

As described in Section 2.2.2, the aqueous extracts of the RSO + 2LYS sample were also studied by ^1^H NMR. In the corresponding spectra, shown in Figure 4A, the presence of various signals can be observed, most of them due to lysine (see Table S2). It is noteworthy that while LC–MS analysis of the aqueous extracts revealed the presence of various lysine adducts with n-alkanals (see Figure 3B–D), most of them seemed to be in too low concentrations to be detected by ^1^H NMR, which is a less sensitive technique. Thus, according to the signal assignments displayed in Table S2, only Nε-formyl-lysine, the most abundant adduct detected by LC–MS, could be identified from day 21 onwards (see signals LA’ and LC’).

With the aim of monitoring the evolution of Nε-formyl-lysine relative to lysine, the ratio between the moles of Nε-formyl-lysine and lysine at each sampling point was calculated from the areas of signals LA’ and LC, respectively (see Figure 4A and Table S2). The equation employed to make this calculation was the following:

N_Lys_ = kA_LC_/2; N_Nε-formyl-lysine_ = kA_LA’_

where N is the number of moles of each compound, k the proportionality constant between the area of the ^1^H NMR signal and the number of protons that generate it, and A_LC_ and A_LA’_ the respective areas of signals LC and LA’.

The evolution of this ratio throughout the RSO + 2LYS sample oxidation process, which is shown in Figure 4B, revealed that, in accordance with the results provided by the LC–MS study, the proportion of Nε-formyl-lysine relative to lysine increased as the process advanced. This is especially noticeable between days 22 and 25, when oxidation proceeded at the fastest rate (see Figure 1 and Figure 2).

##### Reaction with More Reactive Aldehydes

Unlike saturated, unsaturated aldehydes and dialdehydes have more reactive sites in their molecules, so a greater variety of reactions can take place between lysine and this type of aldehyde. These include the formation of Schiff bases and Michael additions, among others [16]. Depending on the extent of the reactions, even more complex products can be generated, like different types of polymers [33].

In this regard, LC–MS analysis of the aqueous extracts revealed the presence of new compounds, dissimilar to the aforementioned *n*-alkanal adducts, above all in the later stages of oxidation (mainly days 21 and 22). Some of these compounds have been tentatively identified, taking into account bibliographic data concerning the molecular weight and mass spectrum of lysine adducts with different types of unsaturated aldehydes, as indicated in the supplementary material. The potential aldehyde involved in each of the adducts, their respective detection days, structures, mass spectra base peaks, and rupture ions, together with the bibliographic references used for their tentative identification, are shown in Table 2; their full mass spectra are also given in the supplementary material. According to the data provided by the TICs (Total Ion Chromatograms) obtained with cone potential 20 V, compounds included in Table 2 exhibit as their base peak an ion with a mass matching the molecular weight of certain lysine adducts described in bibliography plus 1, and in some cases another fragment characteristic of lysine (147 or 130). In addition, the spectra obtained with cone potential 35 V show further ions that were useful in tentatively identifying some of the adducts. In summary, seven adducts were identified: two coming from malondialdehyde (MDA), two from (*E*)-2-alkenals, and three from oxygenated α,β-unsaturated aldehydes. Among these kinds of aldehydes, (*E*)-2-alkenals and the oxygenated α,β-unsaturated type were detected in considerable concentrations during the oxidation process of the RSO oil, but their concentrations were markedly reduced in the presence of lysine, especially in the case of the latter (see Figure 2). Hence, this decrease could be due, at least in part, to the reaction of these aldehydes with lysine.

Adducts with MDA. Although MDA, a very reactive bifunctional aldehyde [6], was not specifically detected under the conditions of this study, it is generated in the peroxidation of polyunsaturated acyl groups or fatty acids. However, detailed studies have evidenced that it is mainly generated from polyunsaturated groups with three or more double bonds, while the linoleic ones, which are the most abundant in soybean oil, are considered weak precursors for MDA [34]. Thus, although MDA would not be expected to be generated in very large amounts from soybean oil due to its relatively low level of triunsaturated acyl groups, it is a very reactive molecule, and in fact this is evidenced in this work. With regard to the MDA adducts here found, the one with base peak *m*/*z* 329 (see Table 2) has been tentatively identified as an N,N’-disubstituted 1-amino-3-iminopropene lysine derivative, resulting from the reaction of MDA with two lysine molecules [6], while that with *m*/*z* 201 might correspond to the Schiff base-type adduct Nε-(2-propenal)lysine. It must be noticed that this latter compound was also identified by Shimozu, Hirano, Shibata, Shibata, and Uchida [35] as one of the reaction products of lysine with 4-hydroperoxy-(*E*)-2-nonenal (4-HPNE). According to these authors, it could be possible that HPNE was first converted to MDA, which would then react with lysine. Therefore, 4-HPNE might also be the first precursor of this adduct.

Adducts with (*E*)-2-alkenals. Regarding the two adducts between lysine and (*E*)-2-alkenals (see Table 2), the one with base peak 259 would match with a Michael adduct of lysine with (*E*)-2-heptenal. Michael addition is in fact considered by some authors [36] to be the most likely pathway for the formation of adducts between lysine and (*E*)-2-alkenals. Lysine adducts with (*E*)-2-heptenal were also found by Globisch, Schindler, Kreßler, and Henle [37] during the incubation at 37 °C of lysine with peanut protein; however, the adducts observed by these authors were mainly due to the addition of two molecules of (*E*)-2-heptenal to lysine, which differ from that tentatively reported here. This suggests that the conditions used in the above-mentioned study, concerning for example aldehyde proportion relative to amino acid, might have led to different adducts. 

As for the compound with base peak 241, only detected at the end of the oxidation process (day 22), this has been tentatively identified as Nε-(3-formyl-3,4-dehydropiperidino)lysine (FDP-lysine), which is a product derived from the reaction of lysine with two molecules of acrolein (2-propenal) [6]. Although the molecular weight of this compound also matches that of a Schiff base-type adduct of lysine with (*E*)-2-heptenal, this reaction can be reversed under acid conditions and indeed these exist in the extracts analyzed.

Adducts with oxygenated α,β-unsaturated aldehydes. With respect to the three adducts presumably coming from the reaction of lysine with oxygenated α,β-unsaturated aldehydes (see Table 2), the ones with base peaks 319 and 301 coincide with the only two specific adducts between lysine and 4-HPNE reported to date [35]. The first, Nε-4-hydroxynonanoic acid-lysine (HNA-lysine), has been suggested as resulting from an initial Michael adduct that undergoes an intramolecular oxidation of the aldehyde group to the carboxyl group catalyzed by the hydroperoxide; the second, Nε-4-hydroxy-(2*Z*)-nonenoyl-lysine, is an amide-type adduct, similar to those formed with n-alkanals. With regard to the adduct with base peak 303, this could arise from 4-hydroxy-(*E*)-2-nonenal (4-HNE) as a result of a Michael addition and further stabilization by cyclization to an hemiacetal. Globisch, Kaden, and Henle [39] also analyzed the occurrence of lysine adducts with 4-HNE in protein from toasted peanuts after a previous hydrolysis process, but in that case the compound detected was a 2-pentylpyrrol derivative.

**Figure 5:**
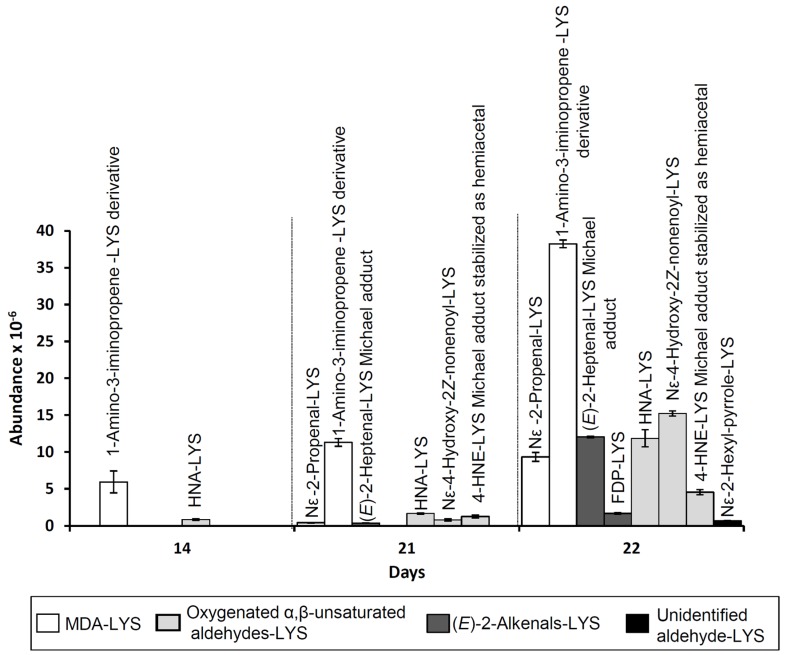
Evolution of the abundance of the mass spectra base peaks of the different adducts between lysine and other aldehydes different from n-alkanals in the aqueous extracts of the RSO + 2LYS sample obtained throughout the oxidation process, determined by LC–MS. All the figures reported are mean values. LYS: lysine; MDA: malondialdehyde; 4-HNE: 4-Hydroxy-(*E*)-2-nonenal.

Figure 5, which shows the evolution of the abundances of the mass spectra base peaks of each of the aforementioned adducts during the oxidation process, reveals that those detected in the first place were the N,N’-disubstituted 1-amino-3-iminopropene lysine derivative, coming from MDA, and HNA-lysine, generated from 4-HPNE. As was the case of adducts of lysine with n-alkanals (see Section 3.2.4), most of the adducts with α,β-unsaturated aldehydes were detected by LC–MS before these aldehydes were noticed in the ^1^H NMR spectra of the RSO + 2LYS sample (see Figure 2A). It is also worth noting that, although according to that commented above, MDA would not be the most likely aldehyde to be generated in the greatest quantity in RSO + 2LYS oil oxidation, perhaps as a result of its high reactivity the 1-amino-3-iminopropene lysine derivative is that exhibiting the highest abundance.

When it comes to the lysine adducts with α,β-unsaturated aldehydes, it is worth noticing that, although (*E*)-2-alkenals, 4-hydroperoxy-, and 4-hydroxy-(*E*)-2-alkenals were generated in similar proportions during oxidation of RSO oil (see Figure 2B), Figure 5 shows that the adducts with the oxygenated α,β-unsaturated type were more numerous, and in general more abundant, than those with (*E*)-2-alkenals, especially those coming from 4-HPNE; these outcomes seem to reflect the greater reactivity of the oxygenated α,β-unsaturated aldehydes.

Another remarkable feature is the great decrease in oxygenated α,β-unsaturated aldehyde concentration observed by ^1^H NMR (see Figure 2) when compared with the abundance of lysine adducts with this type of aldehyde determined by LC–MS (see Figure 5), which is considerably lower than that of some lysine–alkanal adducts like Nε-formyl-lysine (see Figure 3). In this regard, it should be noticed that the adducts detected by this latter technique were only those generated between lysine and volatile aldehydes, but lysine adducts with aldehydes supported on acyl groups would not be taken into account.

##### Pyrrolization reactions

Some oxidation products can lead to the generation of compounds with a pyrrolic structure when reacting with proteins and amino acids. This is the case of some oxygenated α,β-unsaturated aldehydes such as 4,5-epoxy-2-alkenals and 4-hydroxy-(*E*)-2-alkenals [33,39], and also of long-chain epoxy-keto-enes with a 4,5-epoxy-1-oxo-2-pentene system, as commented in Section 3.2.4. Taking into account that, depending on the mechanism involved in the formation of this type of compound, this process can be accompanied by the concomitant generation of saturated aldehydes [30], the occurrence of this type of reaction could contribute to explain the slightly higher concentration of n-alkanals observed during the oxidation process in the system containing lysine than in the reference oil (see Figure 2B). In this sense, among the compounds detected in the LC–MS chromatograms of the aqueous extracts obtained from the RSO + 2LYS sample throughout oxidation, a compound with a pyrrolic structure has also been tentatively identified in the light of its mass spectrum, considering data from Miyashita and coworkers [38]: an Nε-2-hexyl-pyrrole lysine derivative (see Table 2 and Figure 5). As far as we know, this compound has not been previously described either in food or in biological systems, and its precursor remains unknown. Actually, although it has been considered to derive from the reaction of lysine with an aldehyde, another type of precursor should not be discarded. The spectrum and the structure of this compound is displayed in the supplementary material.

##### Polymerization Reactions

In addition to all the issues above commented, it is remarkable that, although not measured, a change in color from yellow to dark orange took place during the oxidation process of the RSO + 2LYS sample. Part of these colored compounds remained in the lipid phase, and another part passed to the acid aqueous extract. Therefore, considering that the reaction between lipid oxidation compounds and amino acids can lead to the formation of colored polymers, either through the spontaneous polymerization of N-substituted hydroxyalkylpyrroles generated in this type of reaction, or through aldol condensation of Schiff bases [40], polymerization might have taken place despite the resulting products not being detected under the conditions of this study.

This hypothetical formation of colored polymers could help to explain some of the observations made in this work, such as: i) the lower degradation extent of linoleic acyl groups in the presence of lysine in comparison with the reference oil when total oil polymerization is reached (see Figure 1A), since polymers in which lysine is involved might be contributing to oil polymerization, so the oil would totally polymerize with a lower linoleic degradation extent; ii) the apparently low abundance of lysine adducts with unsaturated aldehydes found by LC–MS in contrast to the high reduction of the concentration of this kind of aldehyde in the oil, since especially α,β-unsaturated aldehydes can take part in polycondensation reactions, leading to color development to a greater extent than in the saturated ones [41]; and iii) the apparently low decrease in lysine abundance throughout most of the oil oxidation process (see Figure 3A), since lysine might eventually cleave from the polymer, thus restoring the lysine content [40].

### 3.3. Analysis of the Potential Role of Lysine–Aldehyde Adducts on the Antioxidant Effect Observed

According to Alaiz, Zamora, and Hidalgo [42], some of the products coming from the reaction of lysine with aldehydes such as (*E*)-2-octenal or 4,5-epoxy-alkenals can exhibit antioxidant activity in soybean oil, so it could be thought that the lysine adducts identified might contribute to the lower oxidation rate of the oil containing lysine. However, it must be taken into account, on the one hand, that this type of study is performed by adding the previously formed compound to the oil, and on the other, that the compounds identified in this work do not match with those for which antioxidant ability is claimed. In fact, N-alkanoyl-lysine adducts, which were practically the only ones detected during the time period when lysine showed its antioxidant effect (the first twenty days), have not been reported to exhibit antioxidant ability. Therefore, a mechanism other than just the reaction of lysine with aldehydes seems to come into play. In this sense, Alaiz and coworkers themselves [43] pointed out that proteins modified by oxidized lipids are able to exert an antioxidant action on edible oils at the same time as they are being produced, independently of the reactions occurring with oxidation products.

## 4. Conclusions

As far as we know, this is the first time that such a detailed study has been conducted on how the co-oxidation of an edible oil and an amino acid affects the evolution of both the oil components and the amino acid itself by combining ^1^H NMR and LC–MS.

This study confirms the findings of a previous work, evidencing that the addition of 2% of lysine to refined soybean oil exerts a clear antioxidant effect by noticeably retarding oil acyl group degradation and the generation of primary oxidation compounds. Consequently, all the secondary oxidation products appear later in the oil with added lysine, where a remarkable reduction in the concentration of toxic oxygenated α,β-unsaturated aldehydes is also noticed.

Regarding lysine evolution, the main lysine derivatives identified by means of the techniques used were adducts of lysine with different types of aldehydes generated throughout the oil oxidation process. This does not mean that other lysine derivatives were not generated.

The most abundant lysine adducts were those with low-molecular-weight n-alkanals and MDA, followed by the ones with α,β-unsaturated aldehydes like 4-HPNE and (*E*)-2-alkenals, and to a lesser degree, with 4-HNE. To the best of our knowledge, this is the first time that several lysine adducts with aldehydes of varying nature have simultaneously been detected in a complex food model system. Moreover, a lysine derivative with pyrrolic structure, not previously described, has also been found.

Although the ability of lysine to trap very reactive aldehydes could constitute a detoxification mechanism in food systems, it should not be forgotten that this reaction implies a reduction in the biological availability of this essential amino acid.

As it was hypothesized in the Introduction section, a relationship between the evolution of lysine and that of the oil has been observed in this work. However, the formation of adducts between lysine and aldehydes, which is the most outstanding finding, does not seem sufficient to explain the antioxidant effect observed, so further studies would be needed to unravel the specific action mechanism of this amino acid.

Finally, the outcomes of this work may serve to highlight the need to perform the studies about the co-oxidation of lipids and proteins in food systems under conditions as similar as possible to those existing during food processing and storage, since this is crucial to select appropriate markers of the reaction between amino acids and lipid oxidation products.

## Figures and Tables

**Table 1:** Lysine adducts with n-alkanals, identified by LC–MS in the aqueous extracts of the RSO + 2LYS sample throughout the oxidation process, together with their retention times (RT), mass spectra base peaks (BP), and other fragments of their mass spectra.

		Cone 20 V		Cone 35 V	
RT (min)	Compound ^1^	BP ^2^	Other Fragments	BP	Other Fragments ^3^
1:94	Nε-octanoyl-lysine	273	147	273	84, 210, 130, 147
2:26	Nε-hexanol-lysine	245	147	245	84, 182, 147, 130
2:36	Nε-pentanoyl-lysine	231	147	231	84, 168, 147, 130
2:53	Nε-butiryl-lysine	217	147	84	217, 147, 154, 130
2:71	Nε-propanoyl-lysine	203		140	203, 84, 130, 147
2:91	Nε-acetyl-lysine	189		126	84, 189, 147, 130
2:99	Nε-formyl-lysine	175	112	112	175, 84, 130, 147
3:45	Nα-propanoyl-lysine	203		84	203
3:71	Nα-acetyl-lysine	189		84	189
3:73	Nα-formyl-lysine	175		175	84

^1^ Nε-: adducts formed at the ε position; Nα-: adducts formed at the α position.^2^ The base peak corresponds to the molecular weight of the compound plus 1.^3^ The underlined ion is characteristic of each Nε-adduct.

**Table 2:** Compounds coming from the reaction of lysine with aldehydes different from n-alkanals, tentatively identified by LC–MS in the aqueous extracts of the RSO + 2LYS sample obtained throughout the oxidation process, together with their detection day, structure, mass spectra base peaks (BP), and other fragments of their mass spectra. The underlined ions are characteristic of each adduct. MDA: malondialdehyde; LYS: lysine; 4-HPNE: 4-hydroperoxy-(*E*)-2-nonenal; 4-HNE: 4-hydroxy-(*E*)-2-nonenal.

				Cone 20 V		Cone 35 V		
Reaction with	Detection Day	Compound	Structure	BP	Other Fragments	BP	Other Fragments	References
MDA								
	14	N,N’-disubstituted 1-amino-3-iminopropene LYS derivative	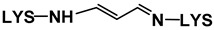	329	147	329	84	[6]
	21	Nε- (2-Propenal)-LYS	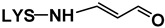	201		84	130, 201, 138	[6]
(*E*)-2-Alkenals								
*(E)-2-Heptenal*	21	(*E*)-2-Heptenal-LYS Michael adduct	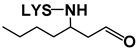	259		259	130, 196	[36]
*Acrolein*	22	Nε- (3-formyl-3,4-dehydropiperidino) lysine (FDP-LYS)	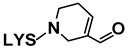	241	130	241	195, 84, 96, 130	[6]
Oxygenated α,β-unsaturated aldehydes								
*4-HPNE*	14	Nε-4-Hydroxynonanoic acid-LYS (HNA-LYS)	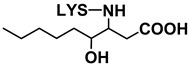	319		319	130	[35]
*4-HPNE*	21	Nε-4-Hydroxy-(2*Z*)-nonenoyl-LYS	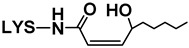	301	147, 130	147	84, 130, 301	[35]
*4-HNE*	21	4-HNE-LYS Michael adduct stabilized as hemiacetal	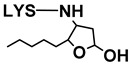	303	147,130	303	84, 130	[6]
Unidentified aldehyde	22	Nε-2-Hexyl-pyrrole-LYS	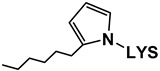	281		197	281, 84, 236, 134	[38]

## Supplementary Materials

The following are available online at https://www.mdpi.com/2076-3921/8/9/326/s1, Table S1: Chemical shifts, multiplicities, and assignments of the ^1^H NMR signals in CDCl_3_ of the main types of triglyceride (TG) protons, and of some oxidation compounds, present in the different soybean oil samples, before and throughout the oxidation process; Table S2: Chemical shifts, multiplicities, and assignments of the ^1^H NMR signals of L-lysine, Nε-formyl-lysine, Nα-acetyl-lysine and Nε-acetyl-lysine in deuterated acid water (0.5 M HCl, pH close to 1.5), obtained from reference compounds; Details of the ^1^H NMR and LC–MS procedures; Standard compounds used for the identification of oxidation products by ^1^H NMR; Equations used for the determination of the molar percentages of the different kinds of oil acyl groups and of the concentrations of oxidation compounds from ^1^H NMR spectral data; Mass spectra and structures of L-lysine and of the different lysine derivatives detected by LC-MS.
