# Supplementary material for: A Dual Perspective of the Action of Lysine on Soybean Oil Oxidation Process Obtained by Combining 1H NMR and LC–MS: Antioxidant Effect and Generation of Lysine–Aldehyde Adducts
Source: Antioxidants (Basel). 2019 Aug 21;8(9):326. doi: 10.3390/antiox8090326 (PMC6770364; doi:10.3390/antiox8090326)

Determination of the molar percentages of the different kinds of oil acyl groups from ^{1}H NMR spectral data

The molar percentages of the several kinds of oil acyl groups were estimated throughout the oxidation process by means of the following equations:

$$Ln\% = 100(A_H/3A) \quad \text{[Equation S1]}$$

$$L\% = 100(2A_G/3A_I) \quad \text{[Equation S2]}$$

$$O\% \text{ (or MU\%)} = 100(A_E/3A_I)\text{-}Ln\%\text{-}L\% \quad \text{[Equation S3]}$$

$$S\% = 100\text{-}Ln\%\text{-}L\%\text{-}O\% \quad \text{[Equation S4]}$$

where A_H and A_G are the areas of the signals of *bis*-allylic protons of linolenic and linoleic groups, respectively (signals "H" and "G" in Table S1). Given that these two signals overlap to a certain extent, the total area corresponding to each of them was calculated using pure trilinolein and trilinolenin (Sigma-Aldrich) as references. A_I, in turn, is the area of the signal of the protons at *sn*-1 and *sn*-3 positions in the glycerol backbone of triglycerides, while A_E corresponds to that of mono-allylic protons (see signals "I" and "E" in Table S1).

Monitoring by ^{1}H NMR of the evolution of RSO and of RSO + 2LYS samples throughout the oxidation process: experimental details and information about acquisition parameters

The weight of each sample aliquot was approximately 0.16 g. These were mixed in a 5 mm diameter tube with 400 μl of deuterated chloroform that contained 0.2% of non deuterated chloroform and a small amount (0.03%) of tetramethylsilane as internal reference.

The acquisition parameters used were: spectral width 5000 Hz, relaxation delay 3 s, number of scans 64, acquisition time 3.744 s and pulse width 90°, with a total acquisition time of 8 min 55 s. The relaxation delay and acquisition time selected allow the complete relaxation of the protons, the signal areas thus being proportional to the number of protons that generate them, making it possible to use them for quantitative purposes. The experiments were carried out at 25 °C, as in previous works [1].

Reference

1. Guillén, M.D.; Ruiz, A. Rapid simultaneous determination by proton NMR of unsaturation and composition of acyl groups in vegetable oils. *Eur. J. Lipid Sci. Technol.* **2003**, *105*, 688-696.

Table S1. Chemical shifts, multiplicities and assignments of the ^{1}H NMR signals in $CDCl_3$ of the main types of triglyceride (TG) protons, and of some oxidation compounds, present in the different soybean oil samples, before and throughout the oxidation process.

Signal	Chemical shift (ppm)	Multi-plicity	Functional group	
			Type of protons	Compound
			**Main acyl groups [2]**	
**A**	0.88	t	$-C\underline{H}_3$	saturated and monounsaturated ω-9 acyl groups
	0.89	t	$-C\underline{H}_3$	linoleic acyl groups
**B**	0.97	t	$-C\underline{H}_3$	linolenic acyl groups
**C**	1.19-1.42	m*	$-(C\underline{H}_2)_n-$	acyl groups
**D**	1.61	m	$-OCO-CH_2-C\underline{H}_2-$	acyl groups in TG
**E**	1.94-2.14	m**	$-C\underline{H}_2-CH=CH-$	unsaturated acyl groups
**F**	2.26-2.36	dt	$-OCO-C\underline{H}_2-$	acyl groups in TG
**G**	2.77	t	$=HC-C\underline{H}_2-CH=$	linoleic acyl groups
**H**	2.80	t	$=HC-C\underline{H}_2-CH=$	linolenic acyl groups
**I**	4.22	dd,dd	$ROC\underline{H}_2-CH(OR')-C\underline{H}_2OR''$	glyceryl groups
**J**	5.27	m	$ROCH_2-C\underline{H}(OR')-CH_2OR''$	glyceryl groups
**K**	5.28-5.46	m	$-C\underline{H}=C\underline{H}-$	acyl groups
			**Oxidation compounds**	
			**Hydroperoxides [3]**	
**a**	8.3-9.0	bs	$-OO\underline{H}$	monohydroperoxide group
			**Conjugated dienic systems [3-5]**	
**-**	5.44	ddd	$-C\underline{H}=C\underline{H}-C\underline{H}=C\underline{H}-$	(*Z*,*E*)-conjugated double bonds associated with hydroxy group in octadecadienoic acyl groups***
**-**	5.66	dd		
**-**	5.97	t		
**b**	6.49	dd		
**-**	5.47	ddm	$-C\underline{H}=C\underline{H}-C\underline{H}=C\underline{H}-$	(*E*,*E*)-conjugated double bonds associated with hydroperoxy group in octadecadienoic acyl groups
**-**	5.76	dtm		
**-**	6.06	ddtd		
**c**	6.27	ddm		
**-**	5.51	dtm	$-C\underline{H}=C\underline{H}-C\underline{H}=C\underline{H}-$	(*Z*,*E*)-conjugated double bonds associated with hydroperoxy group in octadecadienoic acyl groups****
**-**	5.56	ddm		
**-**	6.00	ddtd		
**d**	6.58	dddd		
**-**	5.58	dd	$–C\underline{H}=C\underline{H}–C\underline{H}=C\underline{H}–$	(*E*,*E*)-conjugated double bonds associated with hydroxy group in
**-**	5.71	dd		

**-**	6.03	dd		octadecadienoic acyl groups
**e**	6.18	dd		
			**Epoxides**	
			Epoxy-derivatives	
**f**	2.63 [8]	m	-C**H**O**H**C-	(*E*)-9,10-epoxystearate
**g1**	2.88 [8]	m	-C**H**O**H**C-	(*Z*)-9,10-epoxystearate
**g2**	2.9 [9]	m	-C**H**O**H**C-	monoepoxy-octadecenoate groups
			-C**H**OHC-CH$_2$-CHO**H**C-	diepoxides
**g3**	2.94*****	m	-C**H**O**H**C-	(*Z*)-(12,13)-epoxy-9(*Z*),15(*Z*)-octadecadienoic acid
**h1**	3.10 [9]	m	-CHO**H**C-CH$_2$-C**H**OHC-	diepoxides
			Epoxy-keto-derivatives	
**g4**	2.89 [10]<br>2.90 [11]	td<br>m	-CO-CH=CH-CHO**H**C-	(*E*)-9,10-epoxy-13-keto-(*E*)-11-octadecenoate
**g5**	2.91 [10]	td	-C**H**OHC-CH=CH-CO-	(*E*)-12,13-epoxy-9-keto-(*E*)-10-octadecenoate
**i**	3.20 [10,11]	dd	-CO-CH=CH-C**H**OHC-	(*E*)-9,10-epoxy-13-keto-(*E*)-11-octadecenoate
			-CHO**H**C-CH=CH-CO-	(*E*)-12,13-epoxy-9-keto-(*E*)-10-octadecenoate
			-C**H**OHC-CH=CH-CO-	(*Z*)-12,13-epoxy-9-keto-(*E*)-10-octadecenoate
			-CO-CH=CH-CHO**H**C-	(*Z*)-9,10-epoxy-13-keto-(*E*)-11-octadecenoate
**j1**	3.52 [10]	dd	-CHO**H**C-CH=CH-CO-	(*Z*)-12,13-epoxy-9-keto-(*E*)-10-octadecenoate
**j2**	3.53 [10]	dd	-CO-CH=CH-C**H**OHC-	(*Z*)-9,10-epoxy-13-keto-(*E*)-11-octadecenoate
			Epoxy-hydroxy-derivatives	
**g6**	2.93 [12]	dt	-C**H**OHC-CHOH-CH=CH-	*threo*-11-hydroxy-(*E*)-12,13-epoxy-(*Z*)-9-octadecenoate
**h2**	3.09 [13]<br>3.097 [14]	dd<br>dd	-CHO**H**C-CH=CH-CHOH-	9-hydroxy-(*E*)-12,13-epoxy-(*E*)-10-octadecenoate
			Epoxy-hydroperoxy-derivatives	
**h3**	3.11 [13]	dd	-CHO**H**C-CH=CH-CHOOH-	9-hydroperoxy-(*E*)-12,13-epoxy-(*E*)-10-octadecenoate******
			**Aldehydes [15]**	
**k**	9.49	d	−C**H**O	(*E*)-2-alkenals
**l**	9.52	d	−C**H**O	(*E,E)*-2,4-alkadienals
**m**	9.55	d	−C**H**O	4,5-epoxy-2-alkenals
**n**	9.57	d	−C**H**O	4-hydroxy-(*E*)-2-alkenals
**o**	9.58	d	−C**H**O	4-hydroperoxy-(*E*)-2-alkenals
**p**	9.75	t	-C**H**O	n-alkanals

t: triplet; m: mutiplet; d: doublet; bs: broad signal; *Overlapping of multiplets of methylenic protons in the different acyl groups either in β-position, or further, in relation to double bonds, or in γ-position, or further, in relation to the carbonyl group; **Overlapping of multiplets of the α-methylenic protons in relation to a single double bond of the different unsaturated acyl groups; ***The chemical shifts of the (*Z,E*)- and (*E,Z*)-isomers are practically indistinguishable according to data from reference [6]; **** The chemical shifts of the (*Z,E*)- and (*E,Z*)-isomers are practically indistinguishable according to data from reference [7]; *****Assignment made with the aid of standard compounds; ******δ-Ketols (hydroxy-keto-derivatives) could also contribute to this signal [11].

References

2. Guillén, M.D.; Ruiz, A. *J. Sci. Food Agric.* **2003**, *83,* 338-346.
3. Goicoechea, E.; Guillén, M.D. *J. Agric. Food Chem.* **2010**, *58,* 6234-6245 (hydroperoxides and conjugated (*Z,E*)- and (*E,E*)-hydroperoxy-dienes).
4. Dong, M.; Oda, Y.; Hirota, M. *Biosci. Biotechnol. Biochem.* **2000**, *64,* 882-886 (conjugated (*Z,E*)-hydroxy-dienes).
5. Data taken from Tassignon, P.; De Waard, P.; De Rijk, T.; Tournois, H.; De Wit, D.; De Buyck, L. *Chem. Phys. Lipids* **1994**, *71*, 187-196 (conjugated (*E,E*)-hydroxy-dienes).
6. Kuklev, D.V.; Christie, W.W.; Durand, T.; Rossi, J.C.; Vidal, J.P.; Kasyanov, S.P.; Akulin, V.N.; Bezuglov, V.V. *Chem. Phys. Lipids* **1997**, *85*, 125-134.
7. Chan, H.W.S.; Levett, G. *Lipids,* **1977**, *12*, 99-104.
8. Du, G.; Tekin, A.; Hammond, E.G.; Woo, L.K. *J. Am. Oil Chem. Soc.* **2004**, *81*, 477-480.
9. Aerts, H.A.J.; Jacobs, P.A. *J. Am. Oil Chem. Soc.* **2004**, *81*, 841-846.
10. Lin, D.; Zhang, J.; Sayre, L.M. *J. Org. Chem.* **2007**, *72*, 9471-9480.
11. Gardner, H.W.; Kleiman, R.; Weisleder, D. *Lipids* **1974**, *9,* 696-706.
12. Garssen, G.J.; Veldink, G.A.; Vliegenthart, J.F.; Boldingh, J. *FEBS J.* **1976**, *62,* 33-36.
13. Gardner, H.W.; Weisleder, D.; Kleiman, R. *Lipids* **1978**, *13,* 246-252.
14. Van Os Cornelis, P.A.; Vliegenthart, J.F.G.; Crawford, C.G.; Gardner, H.W. *Biochim. Biophys. Acta,* **1982**, *713,* 173-176.
15. Guillén, M.D.; Ruiz, A. *Eur. J. Lipid Sci. Technol.* **2004**, *106*, 680-687.

Standard compounds used for the identification of oxidation products by ^1H NMR in the oil samples

The standard compounds used for the identification of some oxidation products from the ^1H NMR spectra were the following: (*E*)-2-hexenal, (*E*)-2-heptenal, (*E*)-2-decenal, (*E,E*)-2,4-hexadienal, (*E,E*)-2,4-heptadienal, (*E,E*)-2,4-decadienal and 4,5-epoxy-(*E*)-2-decenal, acquired from Sigma-Aldrich, 4-hydroxy-(*E*)-2-nonenal, 4-hydroperoxy-(*E*)-2-nonenal and *trans*-12,13-epoxy-9-keto-10(*E*)-octadecenoic acid, purchased from Cayman Chemical (Ann Arbor, MI, USA), and *cis*-(12,13)-epoxy-9(*Z*),15(*Z*)-octadecadienoic acid, acquired from Cymit Quimica (Barcelona, Spain).

Quantification of oxidation compounds from 1H NMR spectral data in the oil samples

The concentrations of the oxidation products generated from lipid oxidation in sample RSO and in the oil phase of RSO + 2LYS sample throughout the oxidation process were estimated as millimoles per mole of triglyceride (mmol/mol TG). The general equation to carry out this latter determination was the following:

$$[OP] = [(A_{OP}/n)/(A_I/4)]*1000 \quad \text{[Equation S5]}$$

where A_{OP} is the area of the signal selected for the quantification of each oxidation product (OP) and n the number of protons that generate the signal.

To estimate the concentration of the so-called major epoxides (see section 3.1.4), signals between 2.87 and 3.17 ppm approximately were taken together, assuming that the signal at approximately 2.9 ppm corresponds mainly to epoxides contributing with two protons and the one at 3.1 ppm to epoxy-compounds contributing with only one (see Table S1). In the case of the signal at approximately 2.9 ppm, the overlapped area due to the side band of *bis*-allylic protons signals (G and H) must be subtracted.

Monitoring by LC-MS of the evolution of lysine in the aqueous extracts obtained from the RSO + 2LYS sample throughout the oxidation process: analysis conditions and procedures followed for the identification and quantification of lysine and its derivatives

The LC-MS chromatograms of the aqueous extracts of RSO + 2LYS sample were obtained using a Waters Xevo TQD LC-MS equipment. Sample volumes of 10 µl each were injected into an Imtakt, WAA24 Intrada Amino Acid column (100 mm x 2 mm x 3 µm). A discontinuous gradient of solvent A (H_2O containing 0.1% formic acid) and solvent B (acetonitrile containing 0.1% formic acid) was used as follows: 20% B at 0 min, 75% B at 7 min and 99% B at 7.1 min. Mass spectrometric analysis was performed in TIC mode using positive ion chemical ionization (cone potentials 20 V and 35 V).

The identification of some lysine derivatives was achieved by comparing the mass spectra of the compounds detected in the TICs (Total Ion Chromatograms) obtained with cone potentials 20 V and 35 V with those of lysine adducts obtained in the laboratory by making react lysine with n-alkanals (formaldehyde, propanal, butanal, hexanal, heptanal, octanal and nonanal), as in the study conducted by Kawai, Fujii, Okada, Tsuchie, Uchida and Osawa [16]. For this purpose, lysine (50 mM) was incubated with 50 mM of each alkanal in the presence of H_2O_2 (50 mM) in 50 mM sodium phosphate buffer (pH 7.2) at 37 ℃. Furthermore, bibliographic data were also used to tentatively identify other compounds present in the aqueous extracts.

All the identified lysine derivatives were quantified by measuring the area of their corresponding mass spectra base peaks in the chromatograms obtained with cone potential 20 V. The quantifications were made for them to be useful for comparisons between the samples, not to achieve absolute concentrations of each of the compounds formed.

Reference

[16] Kawai, Y.; Fujii, H.; Okada, M.; Tsuchie, Y.; Uchida, K.; Osawa, T. Formation of Nε-(succinyl) lysine *in vivo*: a novel marker for docosahexaenoic acid-derived protein modification. *J. Lipid Res.* **2006**, *47*, 1386-1398.

Table S2. Chemical shifts, multiplicities and assignments of the ^{1}H NMR signals of L-lysine, Nε-formyl-lysine, Nα-acetyl-lysine and Nε-acetyl-lysine in deuterated acid water (0.5 M HCl, pH close to 1.5), obtained from reference compounds.

Signal	Chemical shift (ppm)[1]	Multiplicity[2]	Type of protons	Compound
		L-Lysine and Nε-Lysine adducts		
LA'	7.86	s	-NεH-C**H**O	Nε-formyl-lysine
LB	3.95	t	-Cα**H**	lysine
LB'	3.94	t	-Cα**H**	Nε-formyl-lysine
LB''	3.93	t	-Cα**H**	Nε-acetyl-lysine
LC'	3.09	t	-Nε-C**H$_2$**-	Nε-formyl-lysine
LC''	3.04	t	-Nε-C**H$_2$**-	Nε-acetyl-lysine
LC	2.85	t	-Nε-C**H$_2$**-	lysine
LD	1.93 - 1.74	m	-Nε-CH$_2$-CH$_2$-CH$_2$-**CH$_2$**-Cα	lysine
LD'	1.90 -1.71	m	-Nε-CH$_2$-CH$_2$-CH$_2$-**CH$_2$**-Cα	Nε-formyl-lysine
LD''	1.87 - 1.69	m	-Nε-CH$_2$-CH$_2$-CH$_2$-**CH$_2$**-Cα	Nε-acetyl-lysine
LG''	1.84	s	-NεH-CO-C**H$_3$**	Nε-acetyl-lysine
LE	1.61 - 1.52	m	-Nε-CH$_2$-C**H$_2$**-CH$_2$-CH$_2$-Cα	lysine
LE'	1.46 - 1.37	m	-Nε-CH$_2$-C**H$_2$**-CH$_2$-CH$_2$-Cα	Nε-formyl-lysine
LE''	1.45 - 1.36	m	-Nε-CH$_2$-C**H$_2$**-CH$_2$-CH$_2$-Cα	Nε-acetyl-lysine
LF	1.46 - 1.26	m	-Nε-CH$_2$-CH$_2$-C**H$_2$**-CH$_2$-Cα	lysine
LF'	1.38 - 1.19	m	-Nε-CH$_2$-CH$_2$-C**H$_2$**-CH$_2$-Cα	Nε-formyl-lysine
LF''	1.37 - 1.17	m	-Nε-CH$_2$-CH$_2$-C**H$_2$**-CH$_2$-Cα	Nε-acetyl-lysine
		Nα-Lysine adducts		
LB'''	4.19 - 4.14	m	-Cα**H**	Nα-acetyl-lysine
LC'''	2.86 - 2.78	t	-Nε-C**H$_2$**-	Nα-acetyl-lysine
LG'''	1.87	s	-NαH-CO-C**H$_3$**	Nα-acetyl-lysine
LD'''	1.79 - 1.56	m	-Nε-CH$_2$-CH$_2$-CH$_2$-**CH$_2$**-Cα	Nα-acetyl-lysine
LE'''	1.57 – 1.47	m	-Nε-CH$_2$-C**H$_2$**-CH$_2$-CH$_2$-Cα	Nα-acetyl-lysine
LF'''	1.37 – 1.22	m	-Nε-CH$_2$-CH$_2$-C**H$_2$**-CH$_2$-Cα	Nα-acetyl-lysine

[1] The chemical shifts of the lysine derivatives have been established taking those of lysine as reference; [2] s: singlet; t: triplet; m: mutiplet.

MASS SPECTRA AND STRUCTURES OF LYSINE AND OF THE TENTATIVELY IDENTIFIED LYSINE ADDUCTS

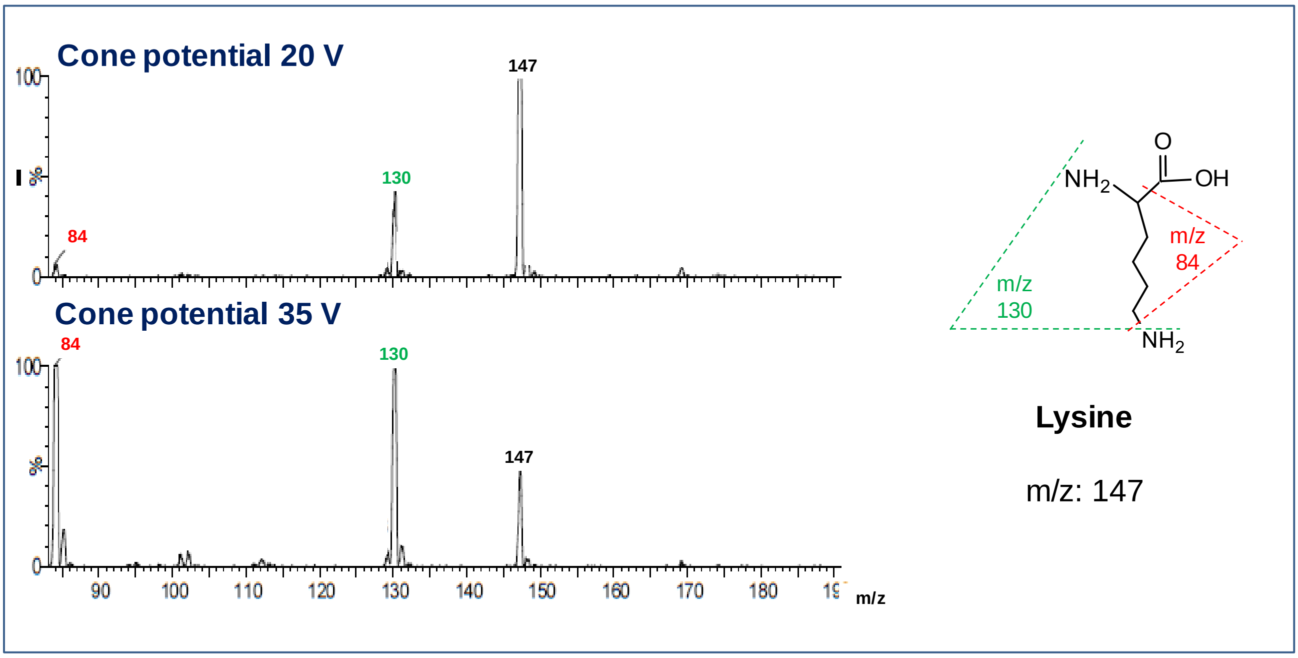

ALKANAL- Nε LYSINE ADDUCTS

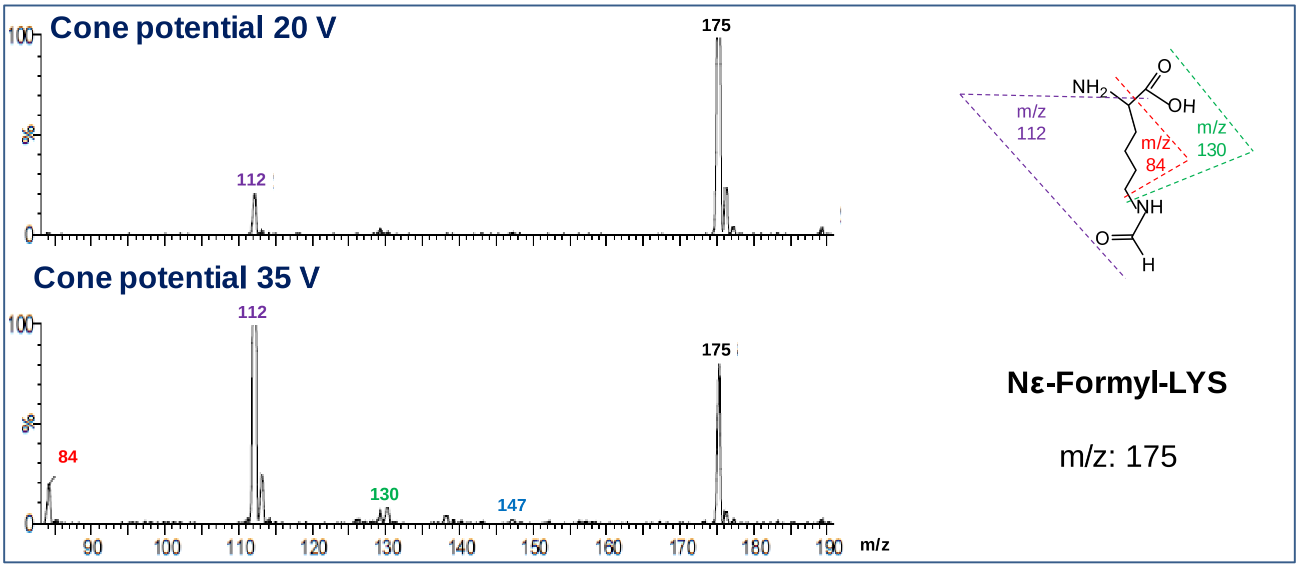

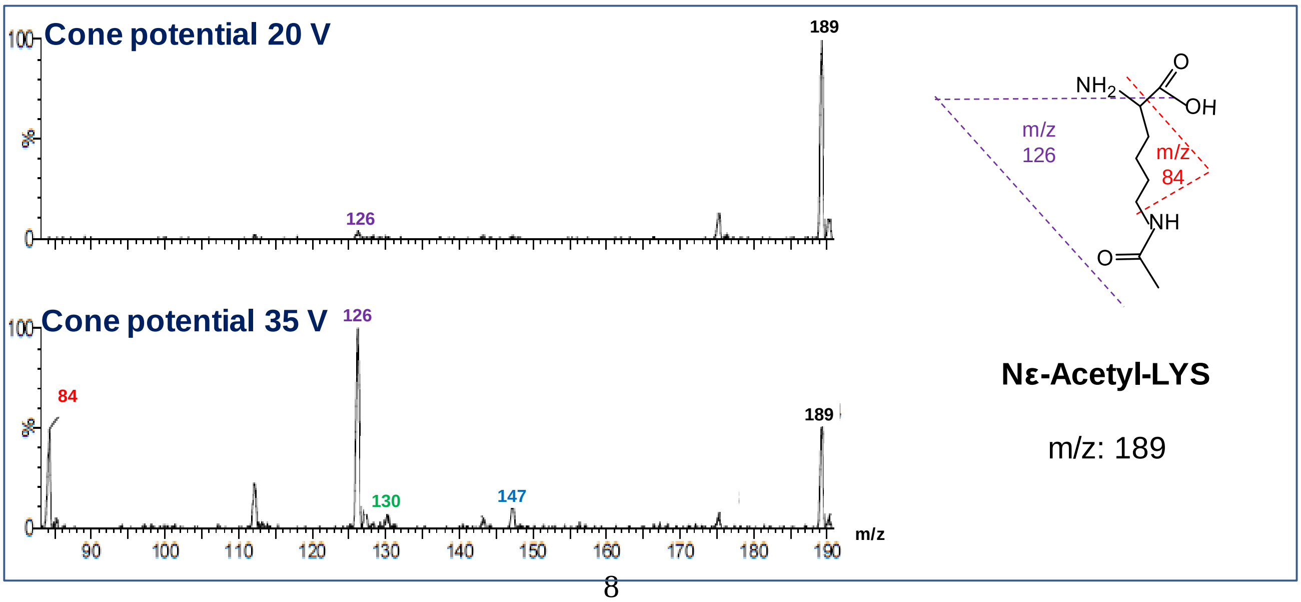

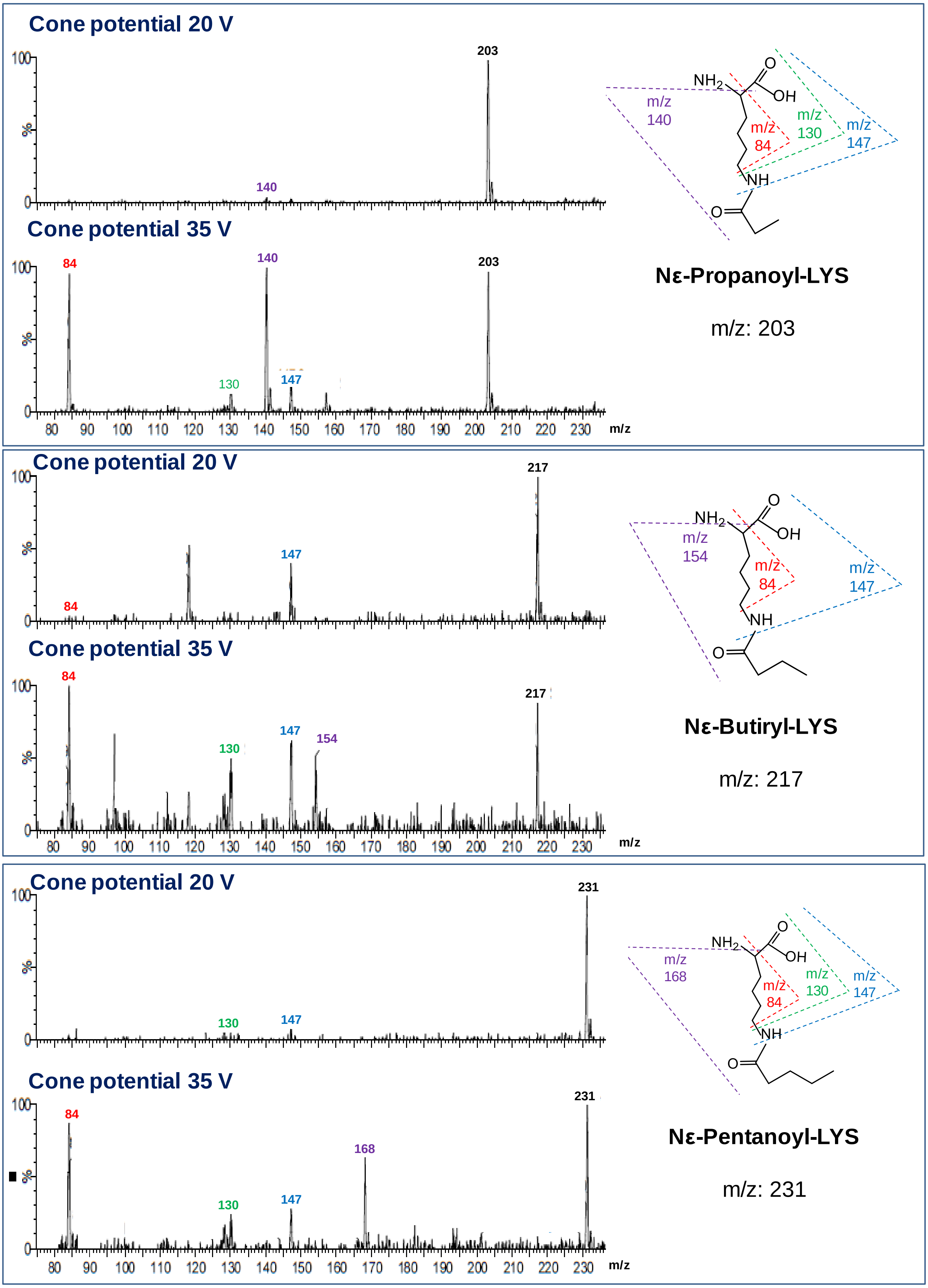
Cone potential 20 V
Cone potential 35 V
100
%
0
203
140
84
130
147
80 90 100 110 120 130 140 150 160 170 180 190 200 210 220 230 m/z
NH2
O
OH
NH
O
m/z 140
m/z 84
m/z 130
m/z 147
Nε-Propanoyl-LYS
m/z: 203
Cone potential 20 V
Cone potential 35 V
217
147
84
130
154
m/z 154
m/z 84
m/z 147
Nε-Butiryl-LYS
m/z: 217
Cone potential 20 V
Cone potential 35 V
231
130
147
84
168
m/z 168
m/z 84
m/z 130
m/z 147
Nε-Pentanoyl-LYS
m/z: 231

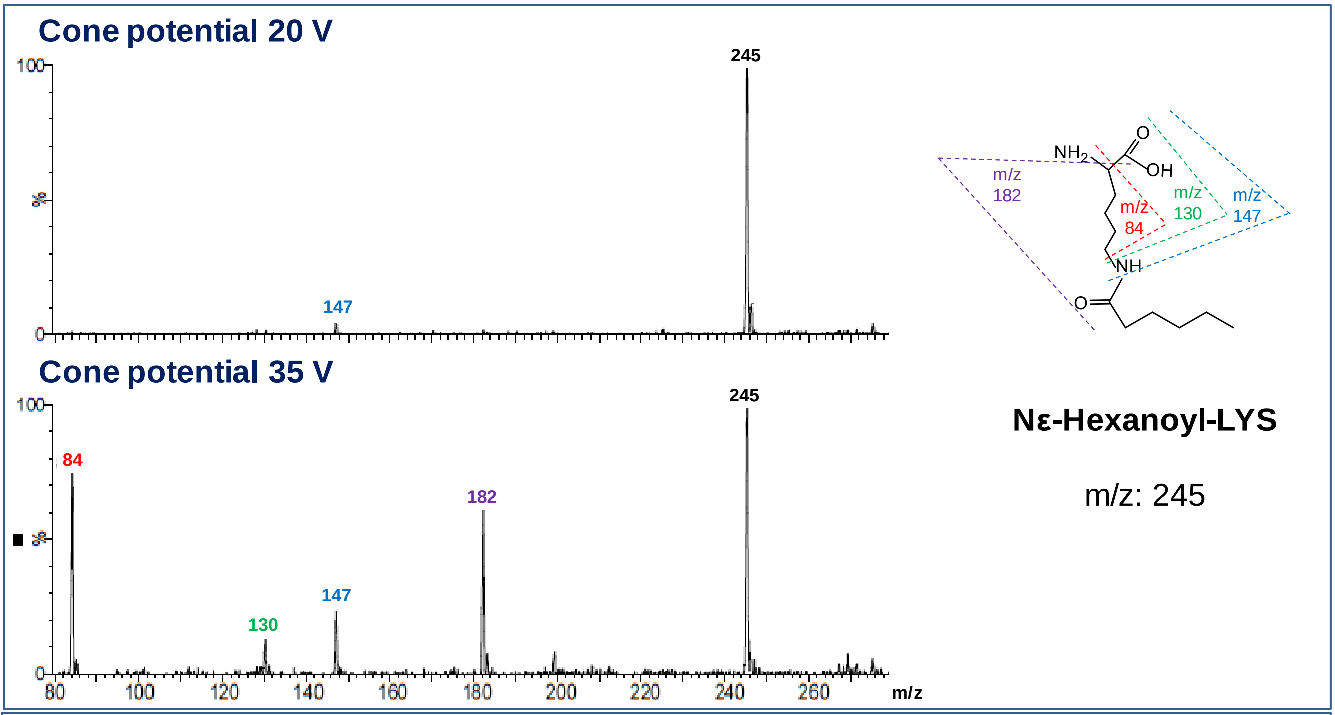
Cone potential 20 V
245
147
Cone potential 35 V
245
84
182
147
130
100
%
0
80
100
120
140
160
180
200
220
240
260
m/z
NH2
O
OH
NH
O
m/z
182
m/z
84
m/z
130
m/z
147
Nε-Hexanoyl-LYS
m/z: 245

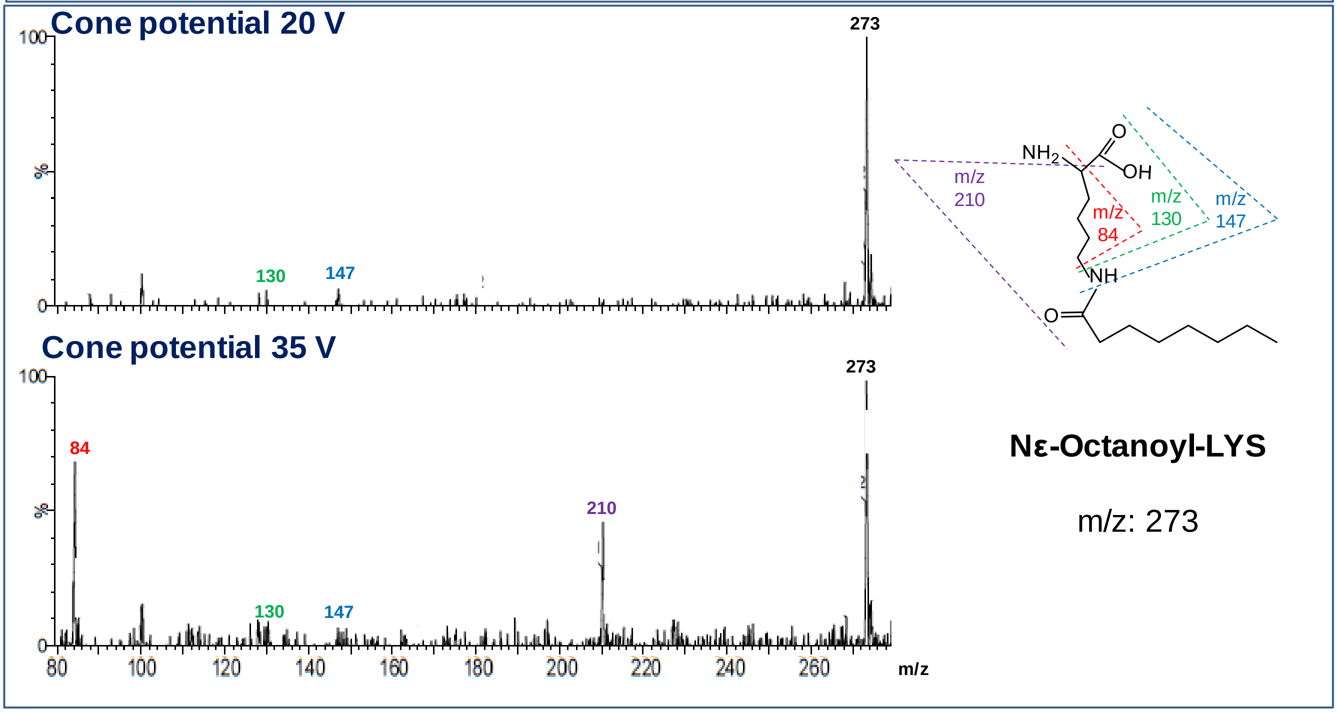
Cone potential 20 V
273
130
147
Cone potential 35 V
273
84
210
130
147
100
%
0
80
100
120
140
160
180
200
220
240
260
m/z
NH2
O
OH
NH
O
m/z
210
m/z
84
m/z
130
m/z
147
Nε-Octanoyl-LYS
m/z: 273

ALKANAL- Nα LYSINE ADDUCTS

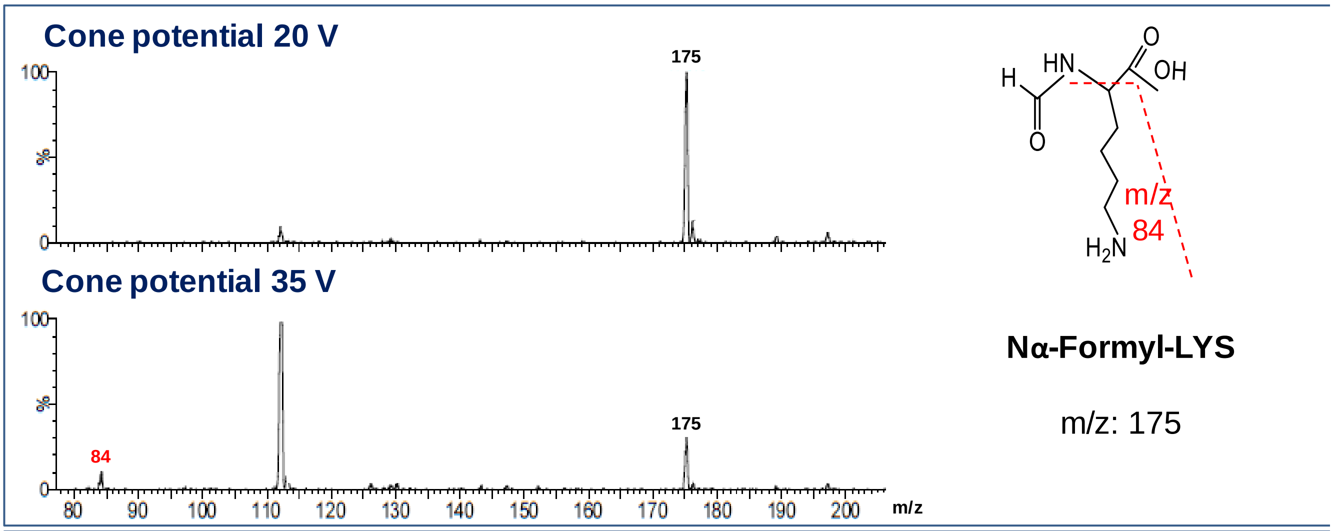

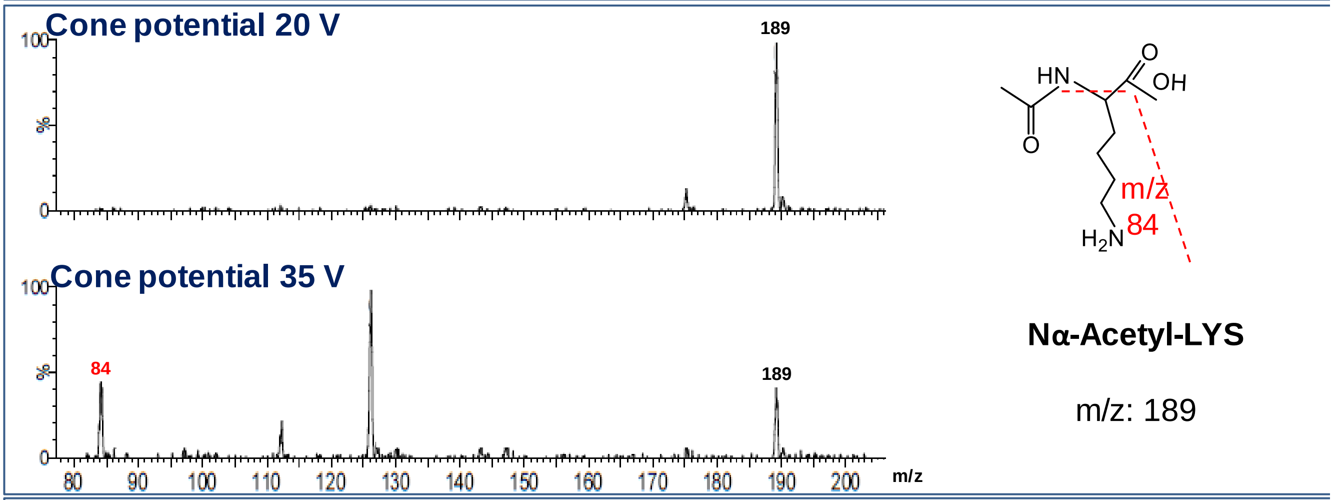

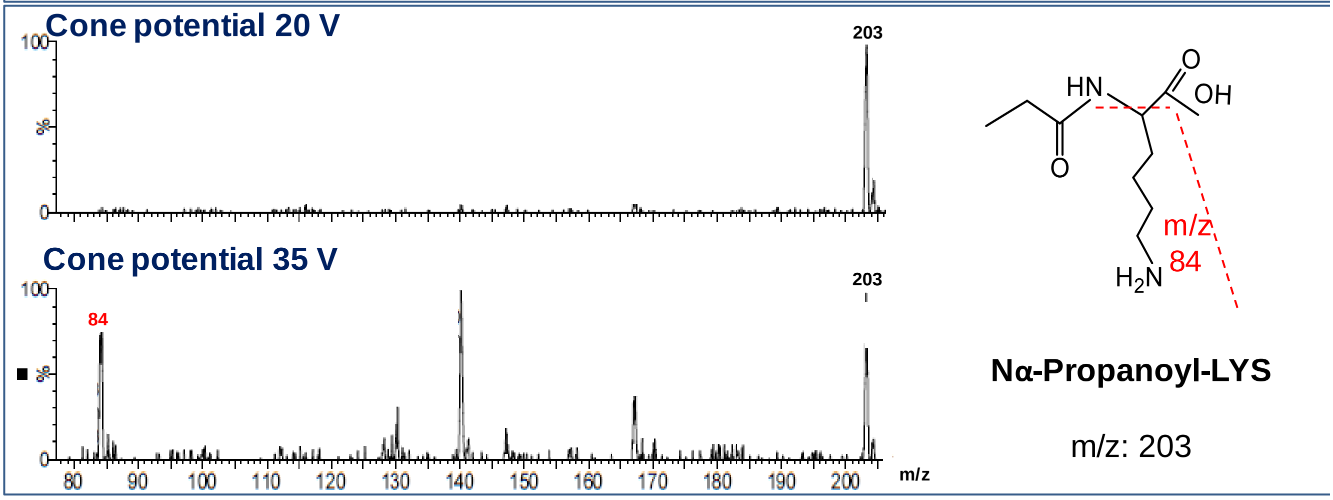

MDA-LYSINE ADDUCTS

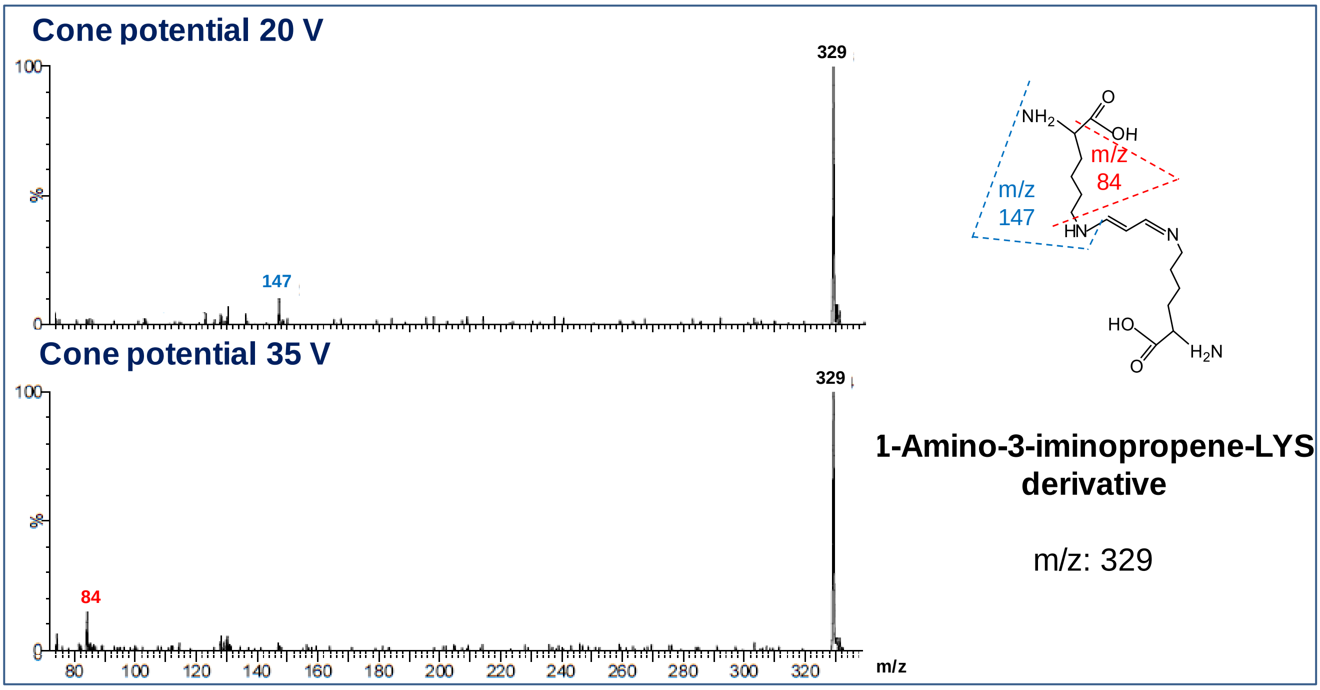

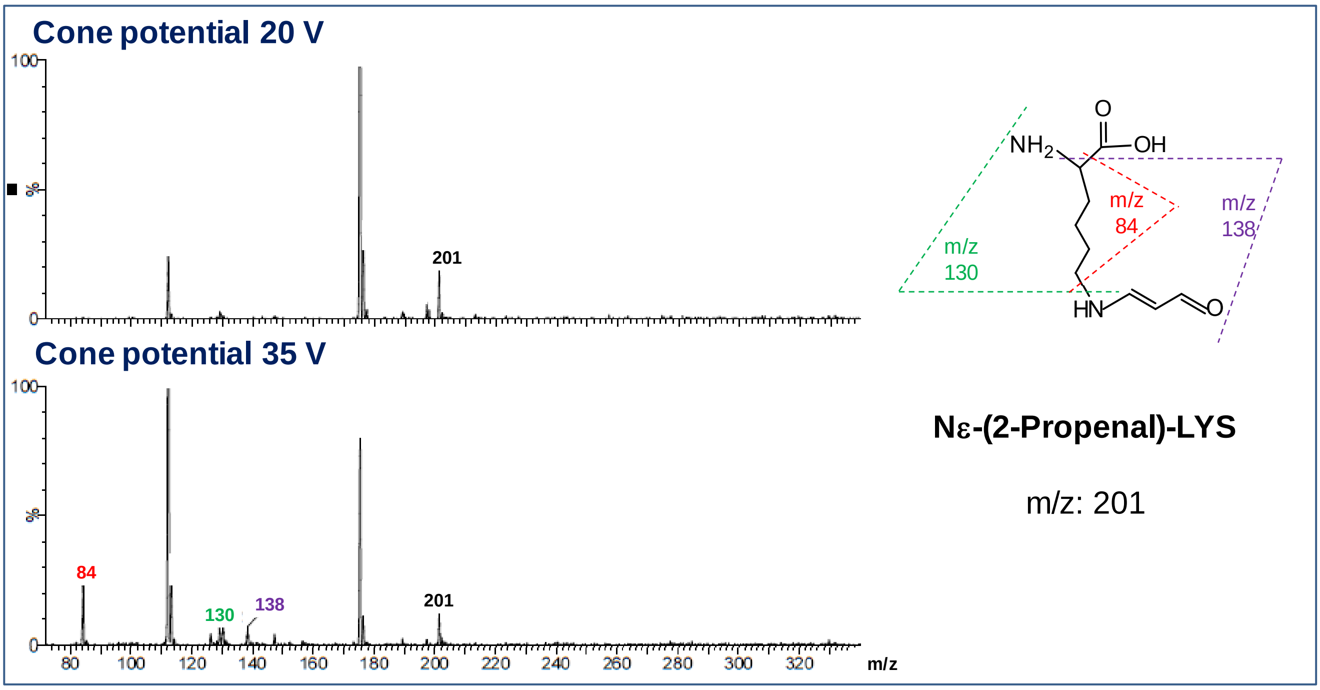

(*E*)-2-ALKENALS-LYSINE ADDUCTS

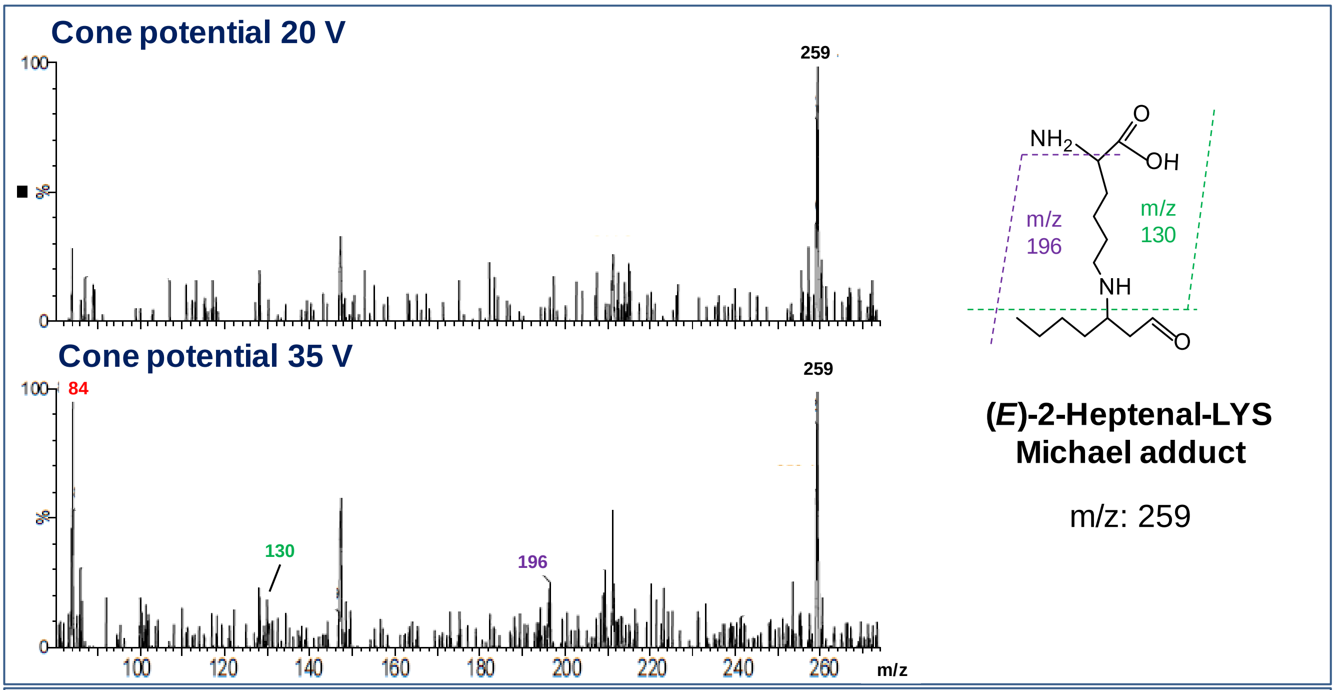

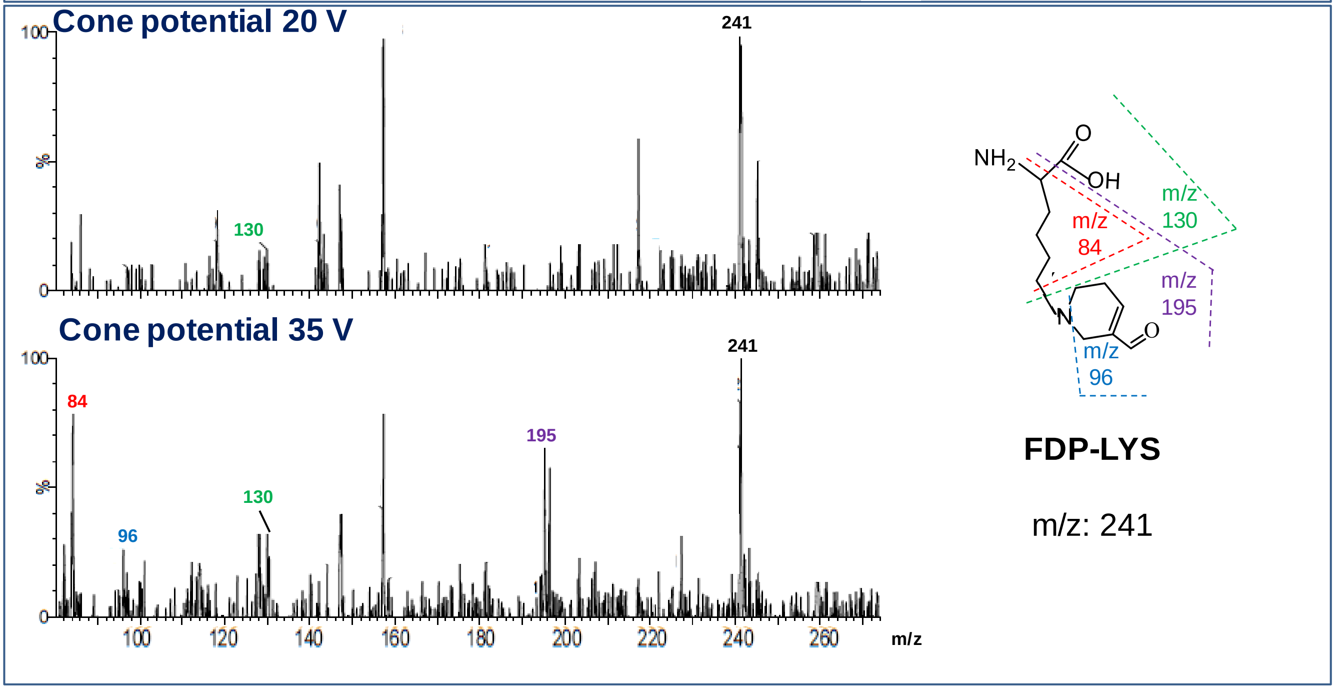

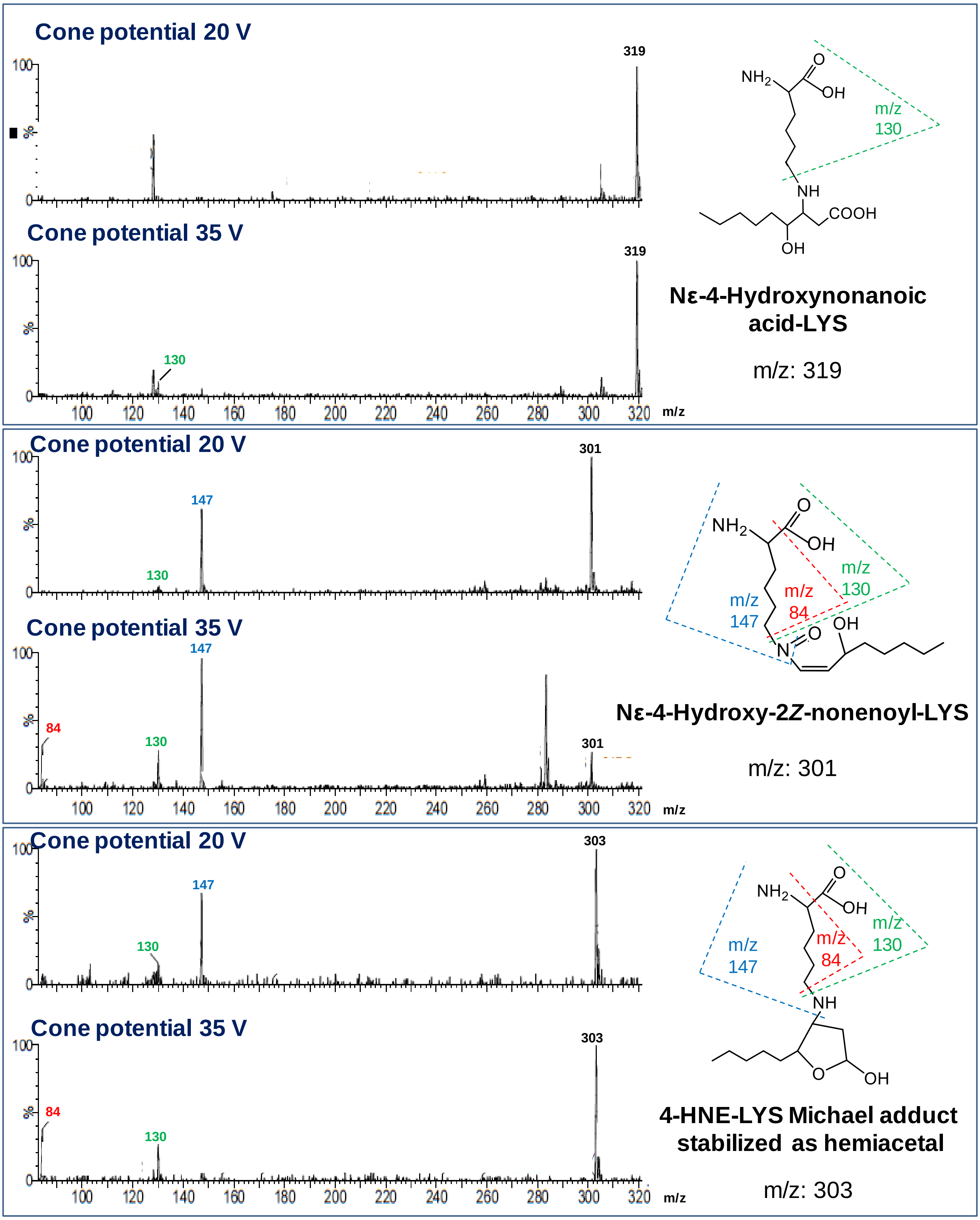
Cone potential 20 V
319
Cone potential 35 V
319
130
100
120
140
160
180
200
220
240
260
280
300
320
m/z
m/z
130
NH2
O
OH
NH
COOH
OH
Nε-4-Hydroxynonanoic acid-LYS
m/z: 319
Cone potential 20 V
301
147
130
Cone potential 35 V
147
84
130
301
m/z
147
m/z
84
m/z
130
N
O
OH
Nε-4-Hydroxy-2Z-nonenoyl-LYS
m/z: 301
Cone potential 20 V
303
147
130
Cone potential 35 V
303
84
130
m/z
147
m/z
84
m/z
130
NH
O
OH
4-HNE-LYS Michael adduct stabilized as hemiacetal
m/z: 303

PYRROLE-TYPE LYSINE ADDUCT

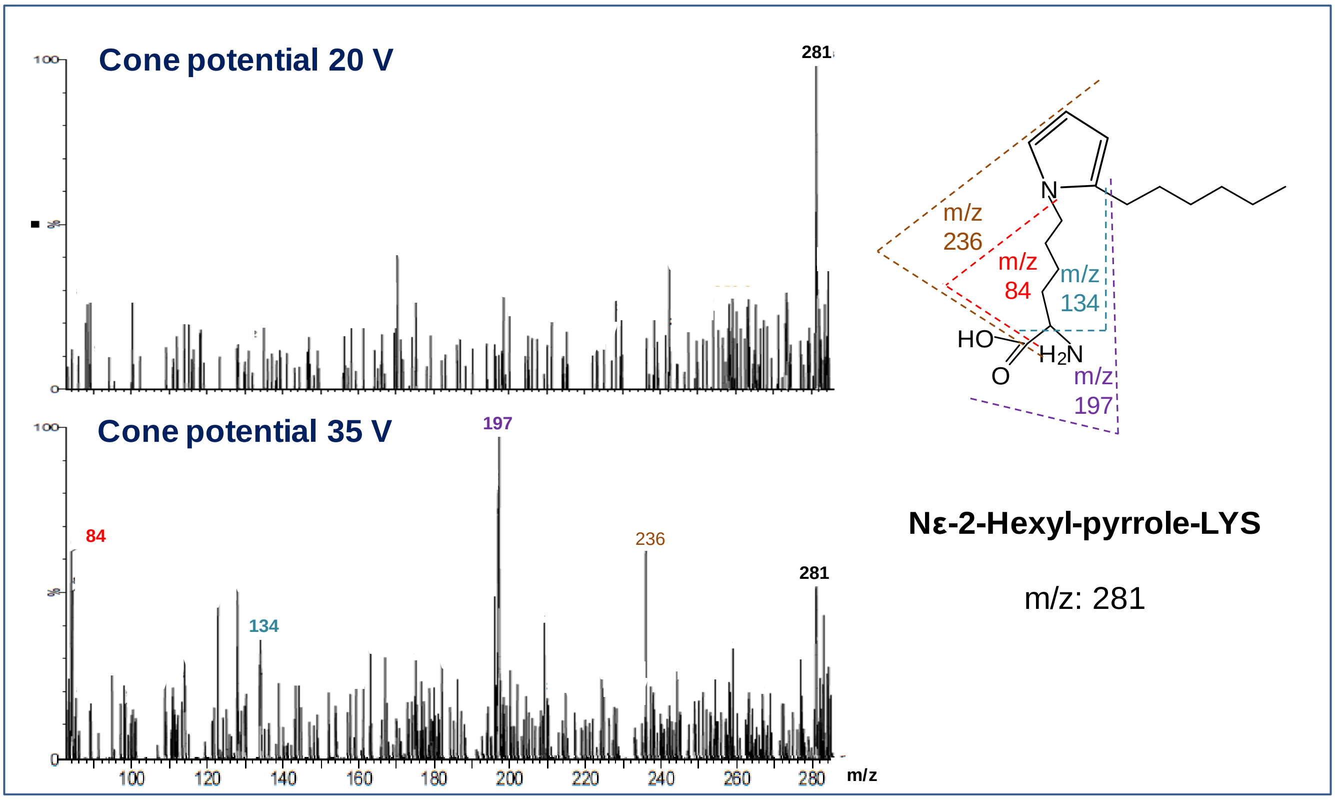

Supplement: Supplementary file 1 [file antioxidants-08-00326-s001.pdf]
